# Discovery of First-in-Class PROTAC Degraders of SARS-CoV-2 Main Protease

**DOI:** 10.1021/acs.jmedchem.3c02416

**Published:** 2024-04-12

**Authors:** Yugendar R. Alugubelli, Jing Xiao, Kaustav Khatua, Sathish Kumar, Long Sun, Yuying Ma, Xinyu R. Ma, Veerabhadra R. Vulupala, Sandeep Atla, Lauren R. Blankenship, Demonta Coleman, Xuping Xie, Benjamin W. Neuman, Wenshe Ray Liu, Shiqing Xu

**Affiliations:** † Texas A&M Drug Discovery Center, Department of Chemistry, Texas A&M University, College Station, Texas 77843, United States; ‡ Department of Biology, Texas A&M University, College Station, Texas 77843, United States; § Department of Biochemistry & Molecular Biology, The University of Texas Medical Branch, Galveston, Texas 77555, United States; ∥ Texas A&M Global Health Research Complex, Texas A&M University, College Station, Texas 77843, United States; ⊥ Department of Biochemistry and Biophysics, Texas A&M University, College Station, Texas 77843, United States; # Institute of Biosciences and Technology and Department of Translational Medical Sciences, College of Medicine, Texas A&M University, Houston, Texas 77030, United States; ∇ Department of Molecular and Cellular Medicine, College of Medicine, Texas A&M University, College Station, Texas 77843, United States; ○ Department of Pharmaceutical Sciences, Irma Lerma Rangel College of Pharmacy, Texas A&M University, College Station, Texas 77843, United States

## Abstract

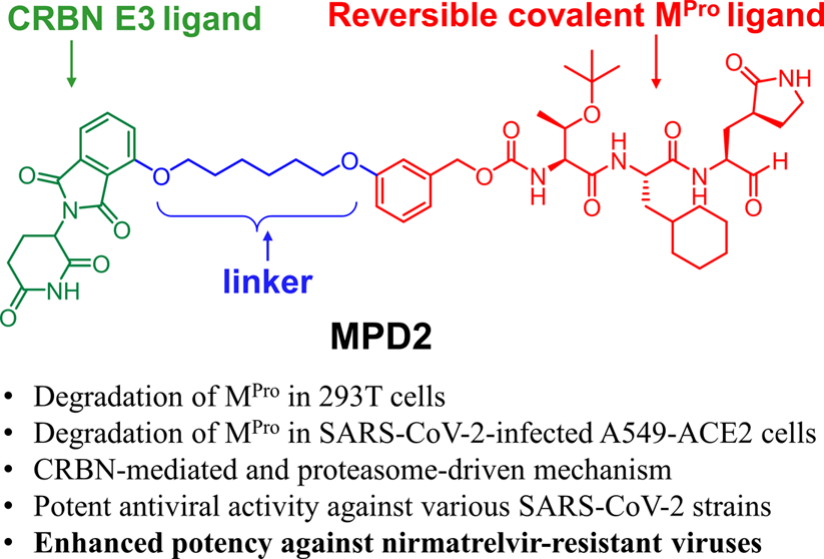

We have witnessed three coronavirus (CoV) outbreaks in the past two decades, including the COVID-19 pandemic caused by SARS-CoV-2. Main protease (M^Pro^), a highly conserved protease among various CoVs, is essential for viral replication and pathogenesis, making it a prime target for antiviral drug development. Here, we leverage proteolysis targeting chimera (PROTAC) technology to develop a new class of small-molecule antivirals that induce the degradation of SARS-CoV-2 M^Pro^. Among them, MPD2 was demonstrated to effectively reduce M^Pro^ protein levels in 293T cells, relying on a time-dependent, CRBN-mediated, and proteasome-driven mechanism. Furthermore, MPD2 exhibited remarkable efficacy in diminishing M^Pro^ protein levels in SARS-CoV-2-infected A549-ACE2 cells. MPD2 also displayed potent antiviral activity against various SARS-CoV-2 strains and exhibited enhanced potency against nirmatrelvir-resistant viruses. Overall, this proof-of-concept study highlights the potential of targeted protein degradation of M^Pro^ as an innovative approach for developing antivirals that could fight against drug-resistant viral variants.

## Introduction

Coronaviruses (CoVs) are a family of single-stranded, positive-sense RNA viruses with a wide-ranging ability to infect mammals, including humans and birds. They are responsible for a spectrum of diseases, including respiratory, hepatic, enteric, and neurologic diseases.^1−5^ Prior to 2003, only a few CoVs such as HCoV-229E and HCoV-OC43^6,7^ were recognized as human pathogens. However, within a span of just two decades, the world witnessed the emergence of three major severe respiratory disease outbreaks: the severe acute respiratory syndrome coronavirus (SARS-CoV) in 2003,^8^ Middle East respiratory syndrome coronavirus (MERS-CoV) in 2012,^9^ and SARS-CoV-2 in 2019.^10−13^ These outbreaks swiftly evolved into significant public health crises. In particular, the ongoing coronavirus disease 2019 (COVID-19) pandemic caused by SARS-CoV-2 has evolved into one of the most formidable public health challenges in human history. According to statistics released from the World Health Organization (WHO) on August 23, 2023, the numbers of confirmed cases and deaths of COVID-19 worldwide have exceeded 770 million and 6.9 million, respectively. The persistent recurrence of CoV pandemics over the past two decades has led scientists to believe that new CoV strains will periodically emerge in human populations. This phenomenon is attributed to various factors, including the high prevalence and widespread distribution of CoVs, their substantial genetic diversity, and the ever-increasing close human–animal interactions in modern society.^14,15^ Given the ongoing COVID-19 pandemic and the potential for future CoV outbreaks, it is imperative to prioritize the development of small-molecule drugs that can be easily distributed as effective CoV antivirals for both treatment and prevention.

The SARS-CoV-2 genome encodes conserved replicase and structural proteins plus several small open reading frames (ORFs) that encode accessory proteins dispensable for virus growth. ORF1ab, the largest ORF, contains overlapping open reading frames that encode polyproteins pp1ab and pp1a.^16^ These polyproteins are cleaved to yield 16 nonstructural proteins (nsps), nsp1–16, through their two viral proteases: papain-like proteinase protein (PL^Pro^, nsp3) and 3C-like (3CL^Pro^, nsp5) that is also called main protease (M^Pro^, nsp5).^17^ These nsps play a pivotal role in viral transcription, replication, proteolytic processing, suppression of host immune responses, and host gene expression. Notably, M^Pro^ is responsible for processing 13 out of the 16 critical nsps that are indispensable for viral replication and packaging. Furthermore, M^Pro^ exhibits a high degree of conservation among various CoVs, making it one of the most attractive drug targets for developing antivirals against COVID-19.^18−20^ M^Pro^ is a cysteine protease with three domains, and its active form is a homodimer comprising two protomers. Each protomer contains a Cys145-His41 catalytic dyad, where cysteine serves as the nucleophile in the proteolytic process.^21^ Consequently, the primary strategy for the development of antivirals has been centered on high-affinity ligands that can bind directly to M^Pro^, thereby inhibiting its enzymatic activities and functions. A significant milestone in M^Pro^ inhibitors was the development of nirmatrelvir, an orally available reversible covalent SARS-CoV-2 M^Pro^ inhibitor developed at Pfizer.^22^ In December 2021, the US FDA granted emergency use authorization for Paxlovid, a combination therapy comprising nirmatrelvir and ritonavir, for the treatment of COVID-19. By May 2023, Paxlovid received full FDA approval for use in high-risk adults and has been granted conditional or emergency use authorization in more than 70 countries worldwide for combating COVID-19. While Paxlovid has generated significant excitement, it is essential to acknowledge several associated issues. These include but are not limited to the potential for serious side effects, drug interactions, the COVID-19 rebound likely due to inadequate drug exposure, and the emergence of drug resistance associated with naturally occurring mutations of SARS-CoV-2 M^Pro^.^23−25^ Given these challenges, there remains a critical need for novel antiviral therapies, particularly those with alternative mechanisms of action that can potentially address these concerns, in the ongoing battle against SARS-CoV-2 and newly emerging CoV pathogens.

Proteolysis targeting chimera (PROTAC) represents an innovative technology for targeted protein degradation in precision medicine.^26−31^ These rationally designed small-molecule PROTACs are heterobifunctional compounds composed of two active ligands interconnected by a chemical linker. One of these ligands is tailored to bind specifically to a protein of interest (POI) while the other selectively engages an E3 ubiquitin ligase. The recruitment of the E3 ligase to the target protein facilitates the formation of a ternary complex, leading to the ubiquitination and ultimate degradation of the target protein.^26−31^ PROTACs offer numerous advantages over traditional occupancy-based inhibitors, including (i) the ability to degrade multidomain proteins, eliminating both enzymatic and nonenzymatic/scaffolding functions;^30^ (ii) flexibility in recruiting targets via various binding sites, even in cases where sustained inhibition is unnecessary; this enables the degradation of “undruggable” targets, using allosteric sites if needed;^32,33^ (iii) the potential to transform weak binders into potent degraders; (iv) a catalytic nature allowing for substoichiometric activity and improved efficacy;^34−36^ (v) enhanced target selectivity achieved through protein–protein interactions between the targeted protein and the recruited E3 ligase;^35−40^ (vi) capability to overcome drug resistance stemming from target protein overexpression by degrading the full-length protein;^41−43^ and (vii) enhanced intracellular accumulation and target engagement.^44^ These distinctive features position PROTAC as a potential game-changing technology in drug discovery that has been widely explored for degrading various disease-causing proteins. Harnessing these advantages, antiviral PROTACs via targeted protein degradation has been considered as a promising strategy for developing next-generation antiviral drugs to combat infectious diseases.^45−48^ The antiviral PROTACs would improve resistance profiles to those of traditional inhibitors.^43,49^ In our ongoing pursuit of COVID-19 drug discovery targeting M^Pro^, we present the design, synthesis, and evaluation of the first series of small-molecule PROTAC degraders targeting SARS-CoV-2 M^Pro^. Our work has led to the identification of MPD2 as a potent degrader of M^Pro^ with a DC_50_ value of 296 nM, demonstrating promising antiviral activity against various SARS-CoV-2 strains and exhibiting enhanced potency against nirmatrelvir-resistant viruses.

## Results and Discussion

### Design of M^Pro^ PROTAC Degraders (MPDs) Based on Reversible Covalent Inhibitors MPI8 and MPI29

**Figure 1:**
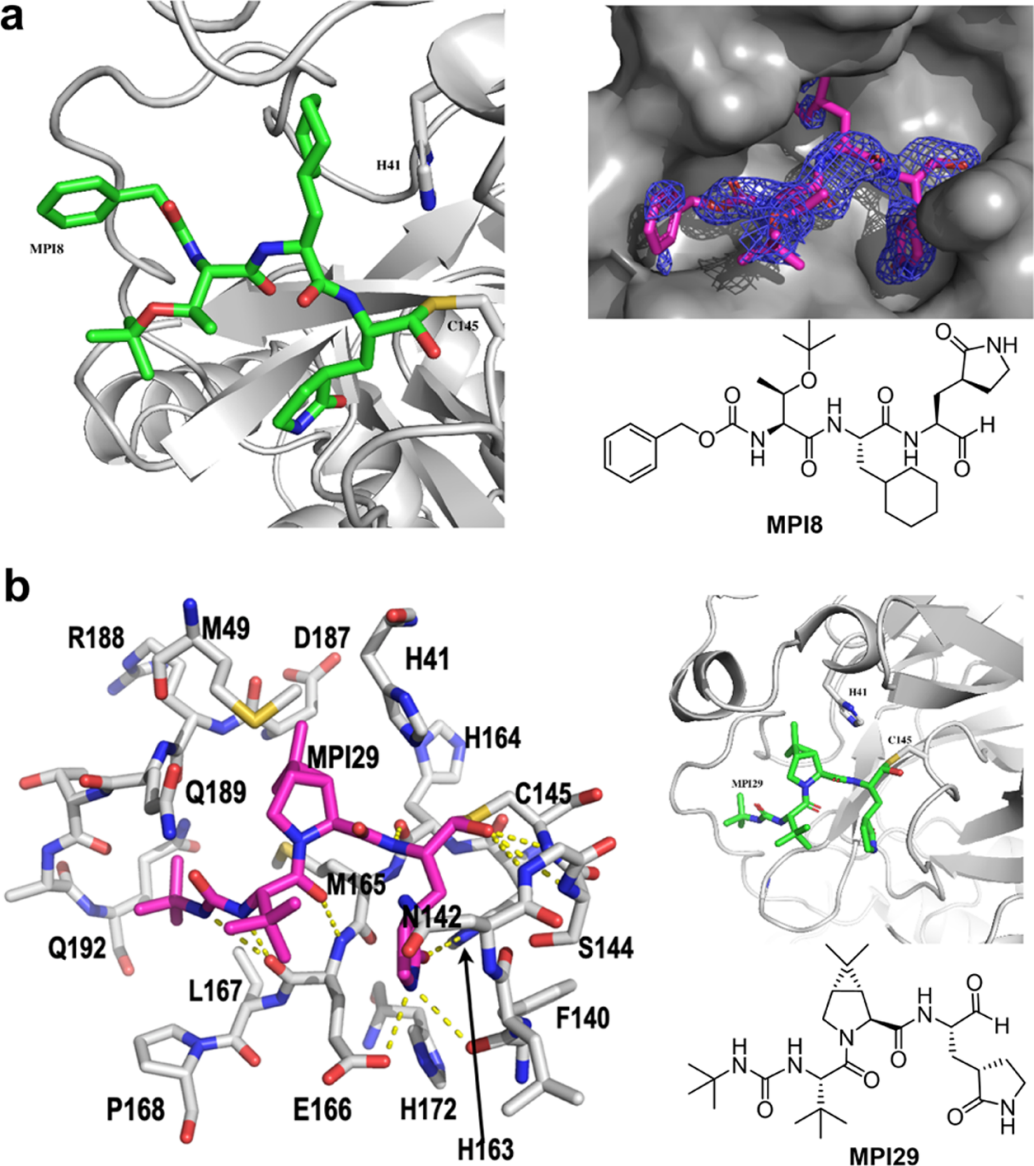
Representative reversible covalent inhibitors MPI8 and MPI29 of SARS-CoV-2 M^Pro^. (a) M^Pro^-MPI8 structure (PDB: 7JQ5).^50^ (b) M^Pro^-MPI29 structure (PDB: 7S6W).^53^

M^Pro^, a protein consisting of 306 amino acids, features an active site comprising four small pockets responsible for binding to the P1, P2, P4, and P1′-3′ residues within a protein substrate.^50^ Crucially, M^Pro^ specifically recognizes a protein substrate containing a strictly P1 glutamine residue. Two active site residues, Cys145 and His41, form a catalytic dyad. An effective strategy for developing M^Pro^ inhibitors involves the conversion of the hydrolytic peptide bond within a substrate into an electrophilic warhead (e.g., aldehyde) to facilitate the formation of a covalent adduct with the catalytic Cys145 of M^Pro^.^20,51^ In 2020, building upon insights from prior medicinal chemistry studies on SARS-CoV M^Pro^ inhibitors,^20,51^ we embarked on the design and synthesis of new reversible covalent inhibitors for SARS-CoV-2 M^Pro^ by (i) employing a more potent β-(*S*-2-oxopyrrolidin-3-yl)-alanine as the fixed P1 residue and enhancing binding due to reduced entropy loss when M^Pro^ engages with the more rigid lactam; (ii) utilizing an aldehyde group as an electrophilic warhead to form a reversible covalent bond with active site Cys145; and (iii) optimizing the P2, P3, and P4 positions for improved potency.^50^ We characterized their enzymatic inhibition and antiviral potency and determined the crystal structures of M^Pro^ bound with these inhibitors. Among these inhibitors, MPI8 emerged with the highest cellular potency against M^Pro^ and highest antiviral activity against SARS-CoV-2.^50,52^ X-ray crystallography analysis of the M^Pro^-MPI8 complex (PDB ID: 7JQ5) revealed that MPI8 fits precisely in the P1- and P2-binding pockets at the M^Pro^ active site (Figure 1a).^50^ Strong van der Waals interactions at the P1- and P2-binding pockets, a number of hydrogen bonds with active site residues, and the covalent interaction with C145 to form a hemithioacetal adduct contribute to the high affinity of MPI8 binding to M^Pro^. The *N*-terminal phenyl group of MPI8 and other inhibitors are not well-defined in the crystal structures, indicating an unfitting size or relatively loosely bound pattern within the P4-binding pocket (Figure 1a).

Boceprevir has been previously explored as a repurposed drug for COVID-19.^54−56^ It serves as an M^Pro^ inhibitor and contains an α-ketoamide warhead, a P1 β-cyclobutylalanyl moiety, a P2 dimethylcyclopropylproline, a P3 *tert*-butylglycine, and an *N*-terminal *tert*-butylcarbamide. In 2020, we designed and synthesized MPI29 by replacing the P1 site of Boceprevir with a 5-oxopyrrolidine-containing residue and altering the warhead to an aldehyde, resulting in an incredibly potent enzymatic inhibitor (IC_50_ = 9.3 nM).^53^ In the M^Pro^-MPI29 complex (PDB ID: 7S6W) as depicted in Figure 1b, MPI29 forms a covalent adduct with M^Pro^ C145 and multiple hydrogen bonds with M^Pro^. Notably, the amide of the lactam at the P1 side chain of MPI29 engages in three hydrogen bonds with M^Pro^ residues including F140, H163, and E166. Additionally, two hydrogen bonds are generated between two backbone amides of MPI29 and M^Pro^ residues involving H164 and E166. The P4 *N*-terminal urea cap of MPI29 employs its two nitrogen atoms to form hydrogen bonds with the backbone oxygen of M^Pro^ E166 (Figure 1b).^53^ Recent studies revealed that the use of reversible covalent inhibitors in PROTAC design is very attractive to combine the benefits of both covalent inhibitors and PROTACs. This represents a new strategy to maintain the catalytic nature of PROTACs while retaining the advantages of covalent PROTACs, such as improved potency, selectivity, and reduced resistance, and with the added advantage of reversibility that could decrease the potential toxicity related to permanent off-target protein labeling.^44,57−60^ Hence, we chose to utilize the reversible covalent M^Pro^ ligands MPI8 and MPI29 for the development of covalent PROTAC degraders for M^Pro^ by taking advantage of the strengths of reversible covalent inhibitors and event-driven PROTAC technology.

**Figure 2:**
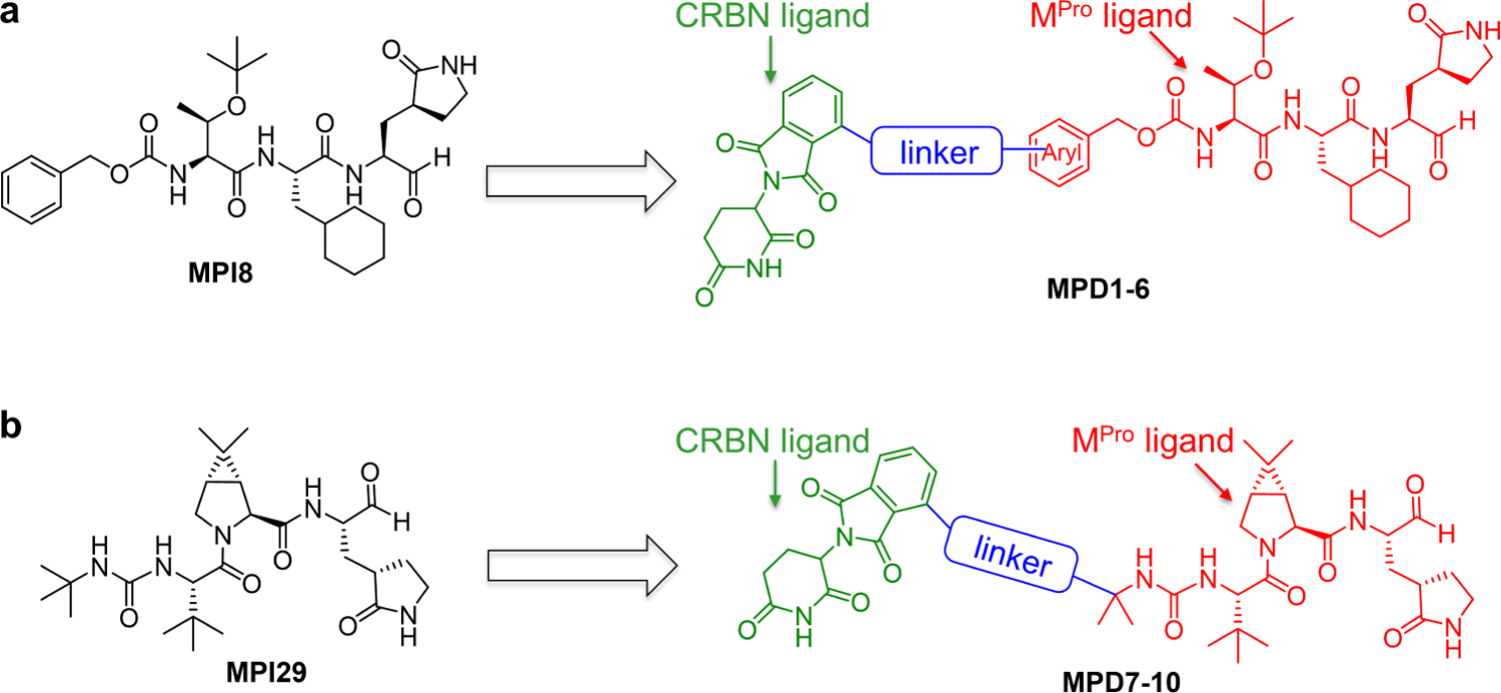
Design of M^Pro^ PROTAC degraders based on the reversible covalent inhibitors MPI8 and MPI29. (a) MPD1–6 via linking a CRBN ligand at the *N*-terminal phenyl group at the P4 site of MPI8. (b) MPD7–10 via linking a CRBN ligand at the *N*-terminal *tert-*butyl group of MPI29.

The crystal structures of the M^Pro^-MPI8 complex (PDB: 7JQ5) and M^Pro^-MPI29 complex (PDB: 7S6W) provided a structural basis to rationally choose a link site for the design of PROTAC degraders. We initiated our research by exploring ubiquitin E3 ligase cereblon (CRBN) ligands as the E3 ligase binders. Drawing insights from crystal structures of the M^Pro^-MPI8 complex (PDB: 7JQ5), it became apparent that the *N*-terminal phenyl moiety of MPI8 exhibited a less defined binding pattern within the P4-binding pocket (Figure 1a). These observations guided the design of a series of M^Pro^ degraders, achieved through the incorporation of a ligand for the CRBN E3 ligase using a diverse array of linkers (Figure 2a): (i) modification of the *N*-terminal phenyl group of MPI8 with different linkers and (ii) substitution of the *N*-terminal phenyl group of MPI8 with a triazole via Cu-catalyzed azide–alkyne cycloaddition with different linkers. Building on crystal structures of the M^Pro^-MPI29 complex (PDB: 7S6W), we identified the *N*-terminal *tert*-butyl moiety of MPI29 at the P4 site as a suitable linker site for introducing the CRBN ligand without interrupting its binding with M^Pro^ (Figure 2b).

**Figure 3:**
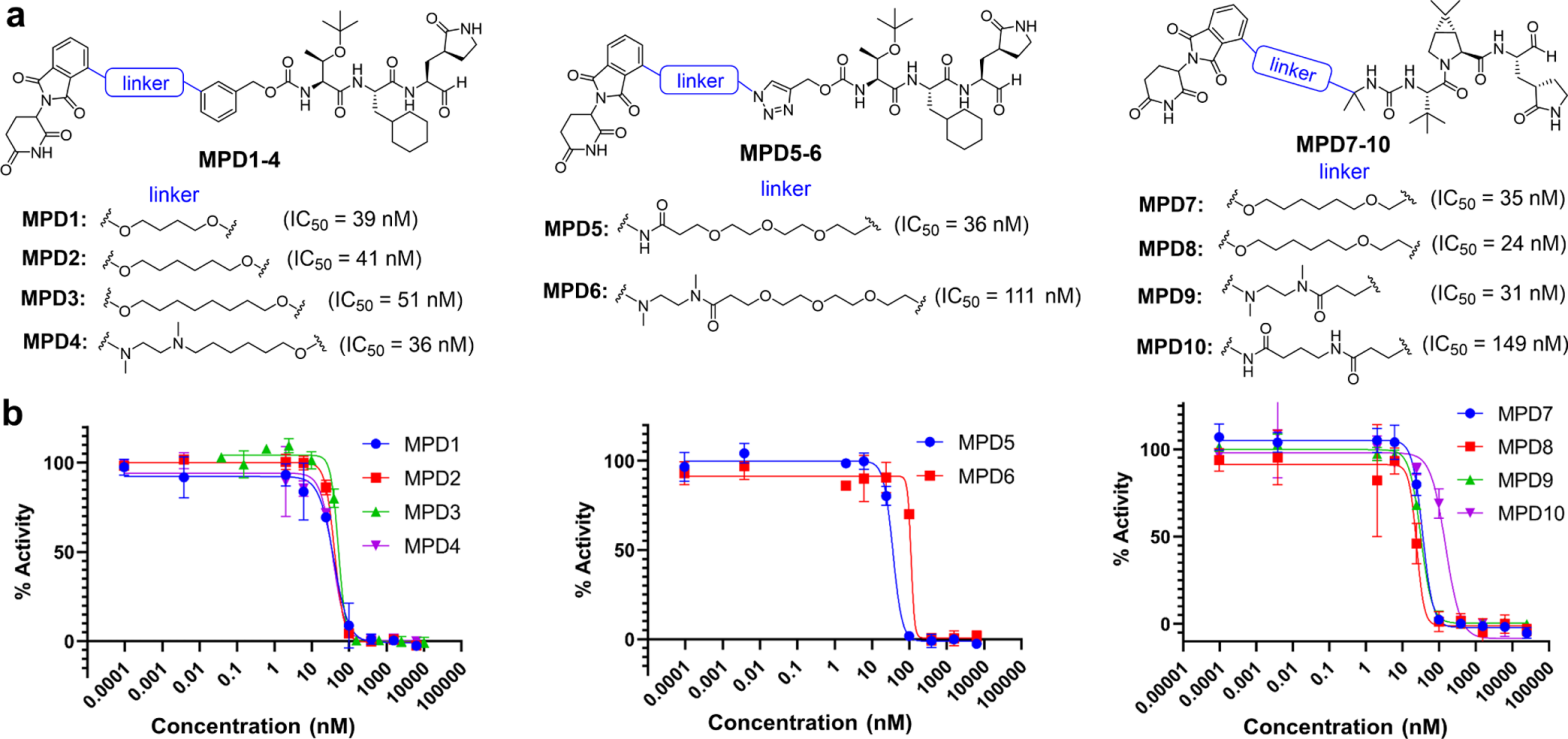
M^Pro^ PROTAC degraders. (a) Chemical structures of M^Pro^ PROTAC degraders MPD1-MPD10. (b) Inhibition curves of MPD1-MPD10 on M^Pro^. Triplicate experiments were performed for each compound. For all experiments, 20 nM M^Pro^ was incubated with an inhibitor for 30 min before 10 μM Sub3 was added. The M^Pro^-catalyzed Sub3 hydrolysis rate was determined by measuring the linear increase of product fluorescence (Ex: 336 nm/Em: 490 nm) at the initial 5 min reaction time.

We synthesized a series of bifunctional small-molecule PROTACs, namely, MPD1-MPD6 based on MPI8 and MPD7-MPD10 based on MPI29, as shown in Figures 3a and S1. The CRBN-binding moieties of MPD1-MPD3 and MPD4 were derived from thalidomide-4-OH and pomalidomide, respectively, two widely used CRBN binders for the PROTAC development.^61^ For MPD5-MPD6, we replaced the *N*-terminal phenyl group of MPI8 with a triazole and introduced pomalidomide CRBN ligand with different linkers via Cu-catalyzed azide–alkyne cycloaddition. For MPI29-based PROTACs MPD7-10, we modified one of the methyl groups of the *N*-terminal *tert-butyl* moiety of MPI29 to introduce thalidomide-4-OH and pomalidomide CRBN ligands (Figure 3a). To assess the inhibitory potency of MPD1-MPD10, we used a previously established protocol that uses Sub3 (Dabcyl-KTSAVLQSGFRKME-Edans) (Figure S2), a fluorogenic peptide substrate of M^Pro^.^62^ In this assay, we preincubated M^Pro^ with a PROTAC molecule for 30 min before Sub3 was added, and the fluorescent product formation (Ex: 336 nm/Em: 490 nm) was recorded in a fluorescence plate reader. As illustrated in Figure 3b, all MPD1-MPD10 retained submicromolar IC_50_ values for the inhibition of SARS-CoV-2 M^Pro^, which indicates that our structure-based design of linker sites works well as there were no obvious interferences with MPDs binding to M^Pro^.

### MPD2 is a Potent M^Pro^ Degrader

**Figure 4:**
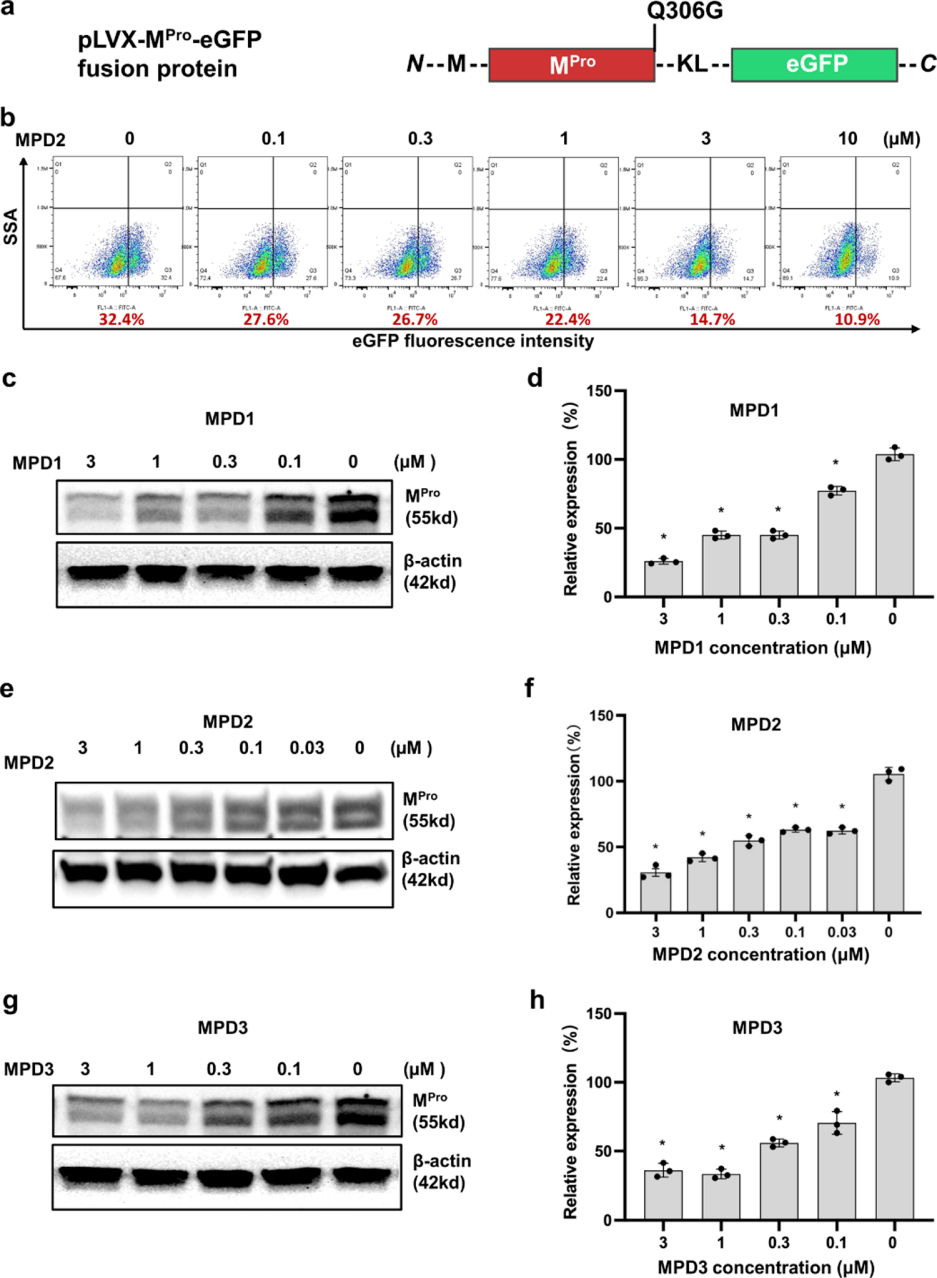
Degradation of M^Pro^ by MPD1-MPD3. (a) Design of M^Pro^-eGFP fusion. (b) Representative flow cytometry analysis of the potency of MPD2 in degrading M^Pro^ in the M^Pro^-eGFP 293T stable cell line. M^Pro^-eGFP cells were evaluated after being treated with different concentrations of MPD2 for 48 h. The percentage of positively expressed M^Pro^-eGFP fusion protein was displayed in red at the bottom of the graph. (c, e, g) The potency of MPD1 (c), MPD2 (e), and MPD1 (g) in degrading M^Pro^ was evaluated in the M^Pro^-eGFP 293T stable cell line by immunoblots after the cells were treated with different concentrations of MPD1, MPD2, and MPD3 for 48 h. Representative immunoblots are shown, and β-actin was used as a loading control in all immunoblot analyses. (d, f, h) The graph presents the normalized protein content in the immunoblots as mean values ± s.e.m. (*n* = 3) in the graph.

To characterize the degradation of M^Pro^ by the PROTAC degraders MPD1-MPD10, we established a cellular system with a stable expression of M^Pro^. For this purpose, we constructed a lentivirus-based system capable of expressing an M^Pro^-eGFP fusion protein (Figure 4a). This system allowed us to generate 293T cells that stably express M^Pro^-eGFP. M^Pro^ natively undergoes cleavage at its *N*- and *C*-terminus and requires a free *N*-terminal end for activation. To maintain the activity of M^Pro^, an *N*-terminal MAVLQ self-cleavage tag was introduced to the fusion protein. Additionally, to prevent the cleavage of *C*-terminal eGFP, C-terminal residue Q306 of M^Pro^ was mutated to Gly. Importantly, the transfection of 293T cells with pLVX-M^Pro^-eGFP shows much less toxicity after multiple rounds of proliferation than another construct we previously reported.^52^ This helps us generate stable cell lines with high levels of eGFP fluorescence for evaluating the M^Pro^ PROTACs. Our flow cytometry results confirmed the expression of the fusion protein, displaying strong fluorescence (Figure 4b). This was further corroborated with Western blot analysis to determine the half-maximal degradation concentration (DC_50_) using an anti-M^Pro^ antibody. Among these M^Pro^ PROTACs, MPD1, MPD2, and MPD3 demonstrated high potency in inducing M^Pro^ degradation in M^Pro^-eGFP stable cell lines. Their DC_50_ values were 419, 296, and 431 nM, respectively (Figure 4c–h). Next, we evaluated the cytotoxicity of MPD1-MPD3 using 293T cells and the MTT assay.^63^ The determined CC_50_ values for MPD1, MPD2, and MPD3 were 25, 120, and 21 μM, respectively (Figure S3). The cytotoxicity curves for these three compounds are presented in Figure S3. Notably, MPD2 exhibited significantly lower toxicity even when compared with the parental MPI8 (CC_50_ = 70 μM). Consequently, MPD2 was selected for further evaluation.

**Figure 5:**
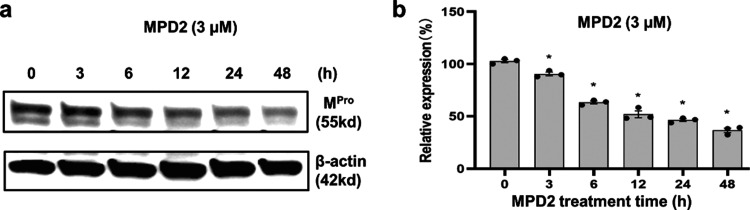
PROTAC degrader MPD2 downregulates the protein levels of M^Pro^ in a time-dependent manner. (a) The time course of MPD2-mediated M^Pro^ degradation was evaluated in the M^Pro^-eGFP 293T stable cell line by immunoblots after the cells were treated with 3 μM MPD2 for various time points as indicated. (b) The graph presents the normalized protein content in the immunoblots as mean values ± s.e.m. (*n* = 3 biologically independent experiments) in the graph.

The induction of M^Pro^ degradation by MPD2 in M^Pro^-eGFP 293T stable cells was rapid and long-lasting. As illustrated in Figure 5a,b, significant degradation was already observed after 6 h of incubation with 3 μM MPD2, and nearly maximal degradation was achieved after 12 h of incubation. To evaluate the *in vitro* metabolic stability of MPD2, we conducted an analysis using human liver microsomes. The determined clearance (CL_int_) value for MPD2 was 36.2 mL/min/kg, resulting in a half-life (*t*_1/2_) of 48 min (Figure S4).

### Mechanism of M^Pro^ Degradation by MPD2

**Figure 6:**
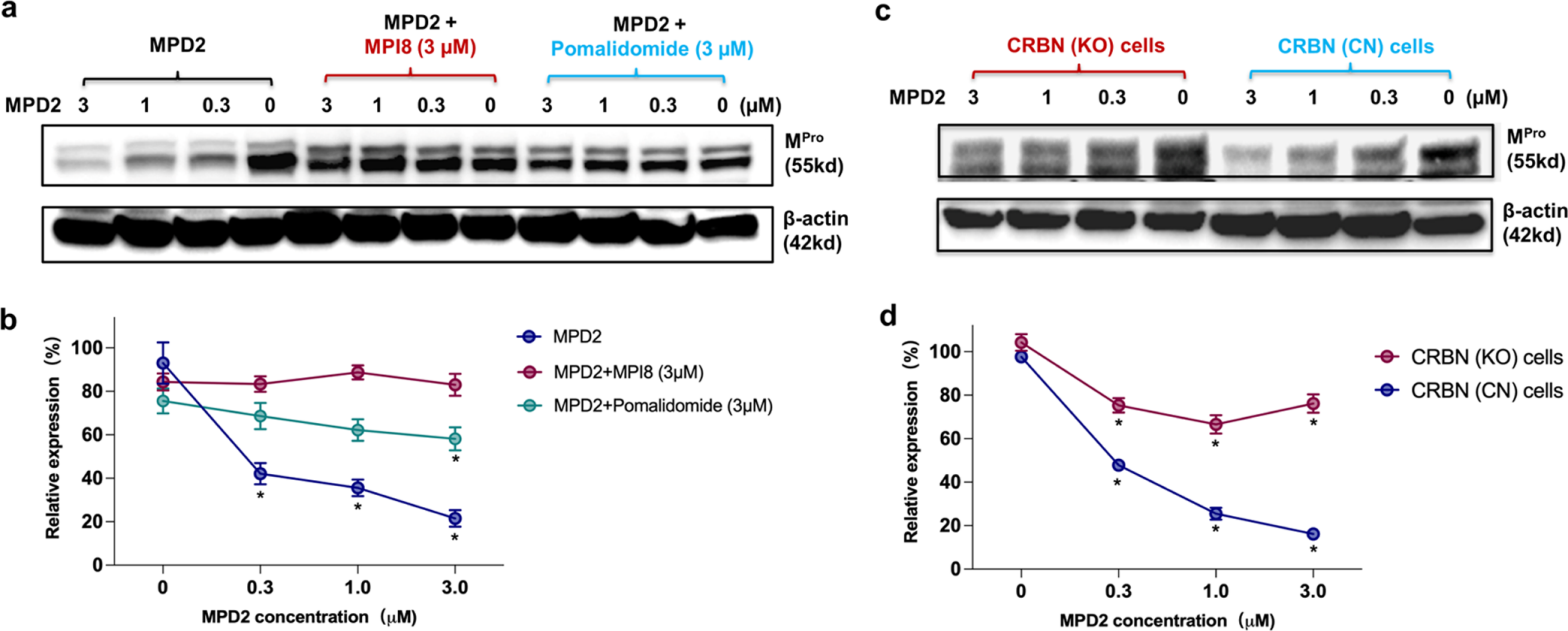
MPD2 degrades M^Pro^ in a ligand- and CRBN-dependent manner. (a) Pretreatment with M^Pro^ ligand MPI8 or CRBN ligand Pomalidomide blocks the M^Pro^ degradation induced by MPD2. The potency of MPD2 in degrading M^Pro^ was evaluated in the M^Pro^-eGFP 293T stable cell line by immunoblots after the cells were treated with different concentrations of MPD2 for 48 h. (b) The normalized protein content in the immunoblots (β-actin was used as a loading control) is presented as mean values ± s.e.m. (*n* = 3 biologically independent experiments) in the titration curved graph. (c) CRISPR knockout of CRBN blocks MPD2-induced M^Pro^ degradation as shown in control (CRBN-CN) and CRBN knockout (CRBN-KO) M^Pro^-eGFP 293T cells. Immunoblots evaluated the potency of MPD2 in degrading M^Pro^ after the cells were treated with different concentrations of MPD2 for 48 h. (d) The normalized protein content in the immunoblots (β-actin was used as a loading control) is presented as mean values ± s.e.m. (*n* = 3 biologically independent experiments) in the titration curved graph.

To validate the mechanism underlying M^Pro^ degradation depending on the concurrent presence of both M^Pro^ and CRBN binding, we conducted competition assay experiments. Initially, the pretreatment of the cells with 3 μM MPI8 (an M^Pro^ ligand) completely blocked the degradation of M^Pro^ by MPD2 (Figures 6a,b and S5). Similarly, the pretreatment of the cells with 3 μM Pomalidomide (a CRBN ligand) resulted in the suppression of M^Pro^ degradation by MPD2 (Figures 6a,b and S5). These studies implied that the degradation of M^Pro^ induced by MPD2 depends on M^Pro^ binding and CRBN binding. To further establish the CRBN dependence of the degradation mechanism of MPD2, we generated CRBN knockout (CRBN-KO) M^Pro^-eGFP 293T cells using the CRISPR editing technique. When comparing the MPD2-induced degradation of M^Pro^ in wild-type CRBN (CN) and CRBN (KO) cells, we observed a significant rescue of intracellular M^Pro^ in cells lacking CRBN (Figures 6c,d and S5). The results of the competition assays with MPI8 and pomalidomide, alongside the CRBN knockout study, collectively affirm that MPD2-induced M^Pro^ degradation relies on both M^Pro^ and CRBN engagement, elucidating a CRBN-mediated mode of action.

**Figure 7:**
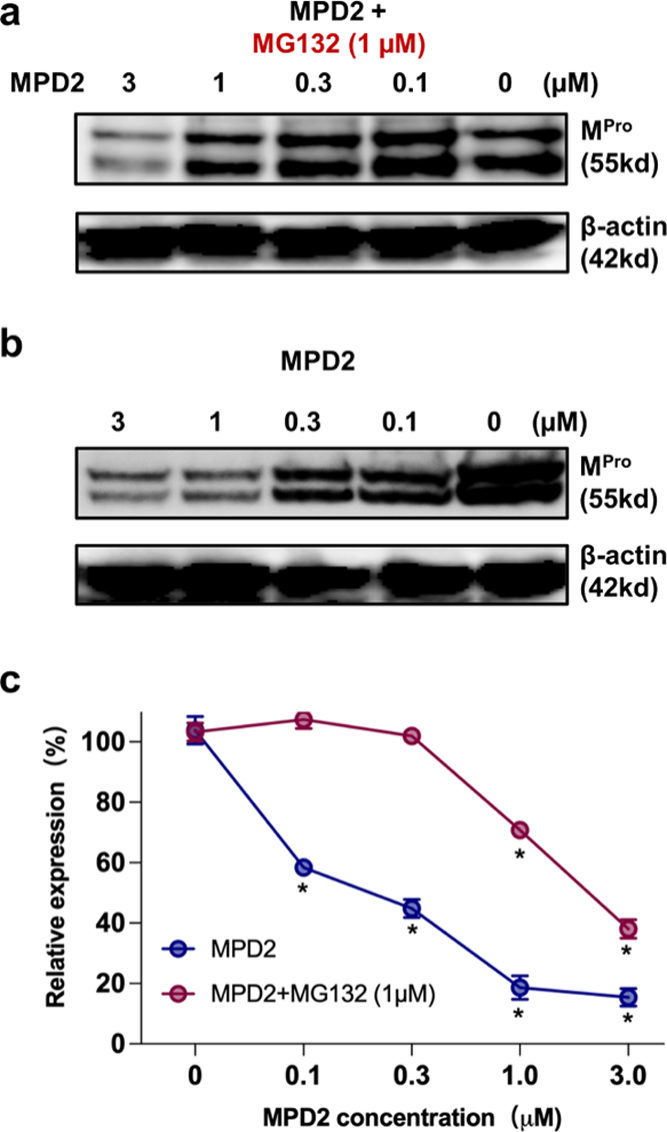
MPD2 degrades M^Pro^ in a proteasome-dependent manner and proteasome inhibition blocks the M^Pro^ degradation by MPD2. (a, b) A representative of 3 immunoblot analyses of M^Pro^ in M^Pro^-eGFP 293T stable cell line after they were either pretreated with the proteasome inhibitor MG132 (1 μM) or pretreated with vehicle for 1 h and then were treated with different concentrations of MPD2 for 48 h. (c) Normalized protein content in the immunoblots (β-actin was used as a loading control) in the titration curved graph.

Moreover, we observed that the proteasome inhibitor MG132 (1 μM) effectively rescued MPD2-induced M^Pro^ degradation. The notable increase in M^Pro^ levels resulting from the cotreatment of cells with MPD2 and MG132 unequivocally validates the indispensability of proteasome activity in the mechanistic degradation of MPD2 (Figure 7). It is essential to note that the presence of MG132 inhibits proteasome activity, thereby impeding the natural ubiquitination process of M^Pro^, which subsequently leads to elevated M^Pro^ levels. Collectively, our studies utilizing CRBN knockout cells, in combination with MPI8, pomalidomide, MG132 and MPD2 treatments, provide compelling evidence that the degradation activity of MPD2 relies on the engagement of M^Pro^, CRBN, and proteasome-mediated degradation. These findings underscore the multifaceted nature of M^Pro^ degradation induced by MPD2, requiring M^Pro^ binding, CRBN binding, and proteasome-mediated processes.

### Antiviral Potency Characterizations

**Figure 8:**
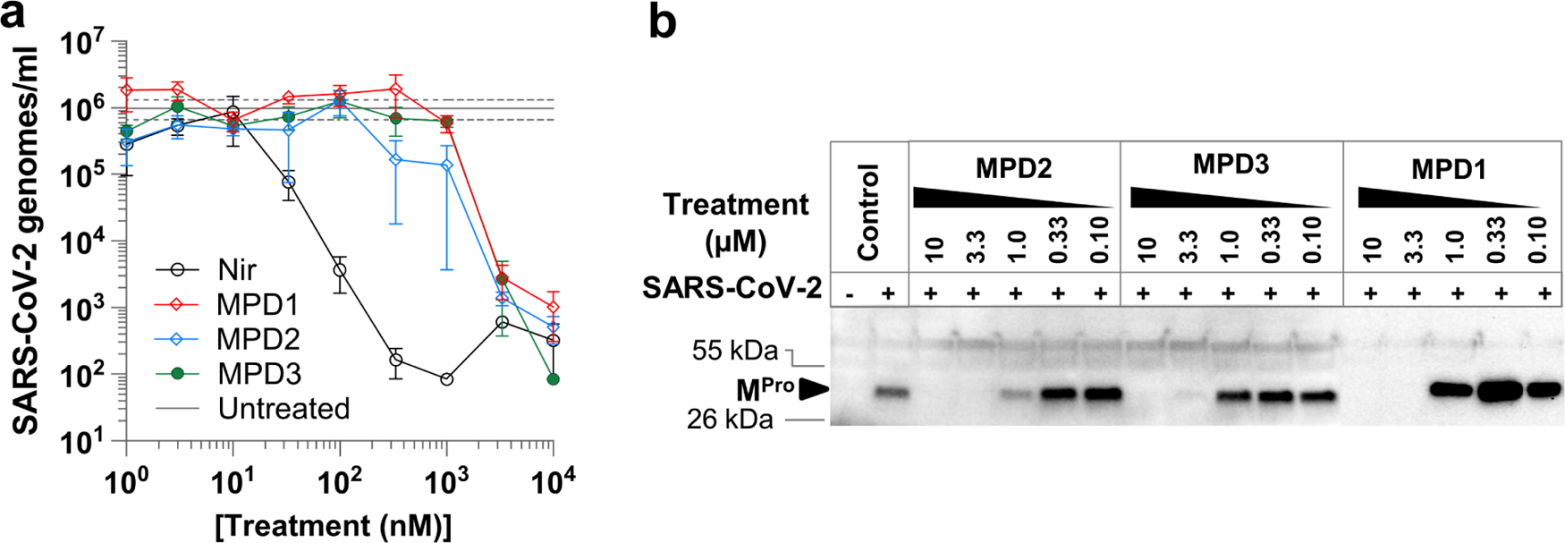
Antiviral effectiveness of PROTACs against the SARS-CoV-2 delta variant. A549-ACE2 cells were inoculated with 0.01 TCID_50_ per cell (a) or 0.1 TCID_50_ per cell (b) SARS-CoV-2 for 1 h and then treated with antiviral. (a) Effect of PROTACs or nirmatrelvir (Nir) treatment on virus growth 48 h after inoculation. (b) Effect of PROTACs treatment on M^Pro^ accumulation 48 h after inoculation with SARS-CoV-2 delta variant. M^Pro^ in cell lysates was detected with monoclonal antibody against 34 kDa M^Pro^.

After successfully validating the degradation of M^Pro^ and understanding the mechanism of degradation induced by the PROTAC degraders, we proceeded to evaluate their antiviral efficacy against SARS-CoV-2 in ACE2^+^ A549 cells infected with hCoV-19/USA/HP05647/2021, an early Delta variant known for its robust growth in cell culture and strong cytopathic effects. MPD1–3 were administered at varying concentrations, and the cells were cultured for 72 h before quantifying SARS-CoV-2 mRNA levels using RT-PCR to determine the antiviral EC_50_ values. MPD1, MPD2, and MPD3 exhibited antiviral EC_50_ values of 1780, 492, and 1160 nM, respectively (Figure 8a). Furthermore, we investigated the impact of PROTAC degrader treatment on M^Pro^ accumulation during SARS-CoV-2 infection. A549-ACE2 cells were inoculated at an approximate multiplicity of 0.1 and subsequently treated with MPD1–3, starting 1 h after inoculation. After 48 h postinoculation, cell lysates were prepared and subjected to blotting using a monoclonal antibody against the 34 kDa M^Pro^. All three degraders, MPD1–3 demonstrated the ability to induce M^Pro^ degradation. Among them, MPD2 emerged as the most potent degrader, which correlates with its highest antiviral activity (Figure 8b).

**Figure 9:**
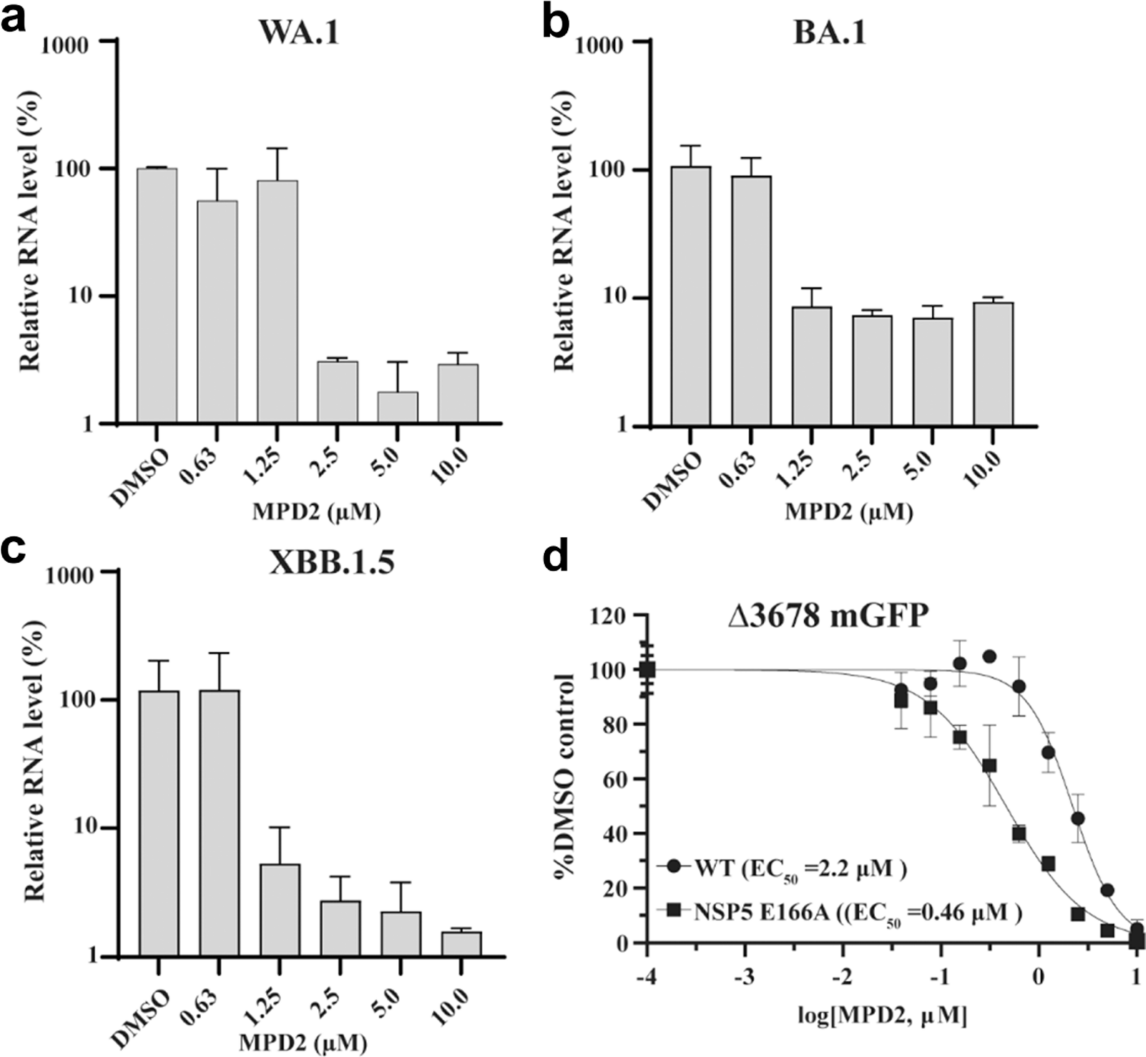
Antiviral Activity of MPD2 against the SARS-CoV-2 variants. MPD2 impedes SARS-CoV-2 strains WA.1 (a), BA.1 (b), and XBB.1.5 (c) infection. A549-hACE2 cells were infected with WA.1 (0.1), BA.1 (0.3), or XBB.1.5 (MOI 0.3) in the presence of various concentrations of MPD2. After 48 h postinfection, supernatant viral RNAs from each treatment dose were quantified and normalized to that of dimethyl sulfoxide (DMSO) controls. (d) MPD2 inhibits the NSP5 E166A mutant. A549-hACE2 cells were infected with the recombinant live-attenuated Δ3678 mGFP or NSP5 E166A mutant and treated with MPD2 for 48 h. mGFP-positive cells from each treatment dose were quantified and normalized to those of DMSO controls. Error bars indicate the standard deviations from duplicates or triplicates.

We further examined the antiviral activity of MPD2 against previous prevalent SARS-CoV-2 strains, including WA.1, BA.1, and XBB.1.5. As expected, at 2.5 μM concentration, MPD2 reduces viral production from infected A549-hACE2 cells by 90% across all three tested strains, demonstrating its efficacy against SARS-CoV-2 variants (Figure 9a–c). Moreover, we assessed the effectiveness of MPD2 against a recombinant SARS-CoV-2 containing an NSP5 E166A mutation, known to confer a 10-fold resistance to nirmatrelvir treatment.^64^ This virus was engineered in the backbone of a live-attenuated BSL-2 SARS-CoV-2 Δ3678 mGFP.^65^ Notably, our results revealed that MPD2 exhibited 5-fold greater potency against the NSP5 E166A mutant virus compared with the wild-type Δ3678 mGFP (Figure 9d). Collectively, our data underscore the significant potential of MPD2 in inhibiting SARS-CoV-2 variants and nirmatrelvir-resistant viruses.

## Discussion

PROTAC is a new technology for targeted therapy in drug discovery. PROTACs are event-driven bifunctional small molecules that simultaneously engage an E3 ubiquitin ligase and a target protein to facilitate the formation of a ternary complex, leading to ubiquitination and ultimate degradation of the target protein. The distinct mechanism of action offers several compelling advantages over traditional inhibitors, including (i) catalytic nature to allow for substoichiometric activity, (ii) recruiting target via any binding site where functional and sustained inhibition is not required, (iii) enhanced target selectivity controlled by protein–protein interactions, (iv) high barrier to resistance, and (iv) abrogating all functions of the target protein and its downstream proteins. These inherent advantages have propelled PROTAC technology into rapid development and wide-ranging applications across various diseases. Numerous small-molecule PROTACs have advanced into clinical trials, particularly in the realm of targeted cancer therapy. While the utilization of PROTAC technology in antiviral research remains relatively limited, and the pool of developed antiviral PROTAC degraders is small, the concept of PROTAC-induced targeted protein degradation is gaining traction as a novel strategy for the development of next-generation antiviral drugs to combat infectious diseases caused by various viruses.^45−48^

In this study, we worked on the design, synthesis, and evaluation of bifunctional M^Pro^ PROTAC degraders MPD1-MPD10 based on the reversible covalent M^Pro^ ligands MPI8 and MPI29 we previously developed. The CRBN-binding moieties of MPD1-MPD10 were derived from the well-established ligands thalidomide-4-OH and pomalidomide. Notably, all MPD1-MPD10 compounds exhibited submicromolar IC_50_ values, underscoring the absence of any discernible interference from the linkers and CRBN E3 ligands with M^Pro^ binding (Figure 3). The M^Pro^ degradation by PROTAC degraders MPD1–MPD3 was determined by a cellular system utilizing the M^Pro^-eGFP fusion protein and Western blot analysis (Figure 4). Our studies revealed that MPD2 effectively facilitated M^Pro^ degradation in M^Pro^-eGFP 293T stable cells, characterized by rapid and enduring effects. To substantiate the degradation mechanism of the M^Pro^ degrader MPD2, we conducted a series of control experiments. Notably, pretreatment with an M^Pro^ inhibitor MPI8 (as a competitor for M^Pro^ binding) or a CRBN E3 ligand pomalidomide (as a competitor of CRBN ligase binding) led to the suppression of M^Pro^ degradation induced by MPD2. These findings strongly implied that MPD2-induced M^Pro^ degradation hinges on both M^Pro^ and CRBN binding. Furthermore, studies involving CRBN knockout cells demonstrated a significant reduction in the degradation potency of MPD2, further affirming a CRBN-dependent mechanism (Figure 6). Our investigation also unveiled that the proteasome inhibitor MG132 could rescue the degradation triggered by MPD2, underscoring the dependence of MPD2’s activity on proteasome-mediated degradation (Figure 7). Taken together, MPD2 has proven to be a potent and effective M^Pro^ degrader that relies on a multifaceted mechanism encompassing M^Pro^ binding, CRBN binding, and proteasome-mediated degradation. Importantly, MPD2 exhibited remarkable antiviral activity against various SARS-CoV-2 variants and successfully degraded M^Pro^ in SARS-CoV-2-infected A549-ACE2 cells (Figures 8–9). Notably, MPD2 exhibited 5-fold greater potency against the NSP5 E166A mutant nirmatrelvir-resistant virus compared with the wild-type Δ3678 mGFP (Figure 9d). Collectively, our data underscore the significant potential of MPD2 in inhibiting SARS-CoV-2 variants and nirmatrelvir-resistant viruses (Figure 9).

In summary, we have developed a series of SARS-CoV-2 M^Pro^ degraders, with MPD2 emerging as a standout lead compound. MPD2 effectively reduced M^Pro^ protein levels in 293T cells, relying on a time-dependent, CRBN-mediated, and proteasome-driven mechanism. Furthermore, MPD2 exhibited remarkable efficacy in diminishing M^Pro^ protein levels in SARS-CoV-2-infected A549-ACE2 cells. MPD2 also displayed potent antiviral activity against various SARS-CoV-2 strains and exhibited enhanced potency against nirmatrelvir-resistant viruses. This proof-of-concept study highlights the potential of leveraging PROTAC-mediated targeted protein degradation as a novel approach to target M^Pro^ for developing antivirals that could fight against drug-resistant viral variants.

## Experimental Section

### Materials

We purchased yeast extract from Thermo Fisher Scientific, tryptone from Gibco, Sub3 from Bachem, HEK293T/17 cells from ATCC, Dulbecco’s modified Eagle’s medium (DMEM) with GlutaMax from Gibco, fetal bovine serum (FBS) from Gibco, polyethylenimine from Polysciences, and the trypsin-ethylenediamine tetraacetic acid (EDTA) solution from Gibco. Chemicals used in this work were acquired from Sigma-Aldrich, Chem Impex, Ambeed, and A2B. Pooled human liver microsome (1910096) was obtained from Xenotech.

### M^Pro^ Expression and Purification

The expression plasmid pET28a-His-SUMO-M^Pro^ was constructed as in a previous study. We used this construct to transform *Escherichia coli* BL21(DE3) cells. A single colony grown on an LB plate containing 50 μg/mL kanamycin was picked and grown in 5 mL of LB media supplemented with 50 μg/mL kanamycin overnight. We inoculated this overnight culture to 6 L of 2YT media with 50 μg/mL kanamycin. Cells were grown to OD_600_ as 0.8. At this point, we added 1 mM IPTG to induce the expression of His-SUMO-M^Pro^. Induced cells were allowed to grow for 3 h and then harvested by centrifugation at 12,000 rpm, 4 °C for 30 min. We resuspended cell pellets in 150 mL of lysis buffer (20 mM Tris-HCl, 100 mM NaCl, 10 mM imidazole, pH 8.0) and lysed the cells by sonication on ice. We clarified the lysate by centrifugation at 16,000 rpm, 4 °C for 30 min. We decanted the supernatant and mixed it with Ni-NTA resins (GenScript). We loaded the resins to a column, washed the resins with 10 volumes of lysis buffer, and eluted the bound protein using elution buffer (20 mM Tris-HCl, 100 mM NaCl, and 250 mM imidazole, pH 8.0). We exchanged buffer of the elute to another buffer (20 mM Tris-HCl, 100 mM NaCl, 10 mM imidazole, 1 mM DTT, pH 8.0) using a HiPrep 26/10 desalting column (Cytiva) and digested the elute using 10 units SUMO protease overnight at 4 °C. The digested elute was subjected to Ni-NTA resins in a column to remove His-tagged SUMO protease, His-tagged SUMO tag, and undigested His-SUMO-M^Pro^. We loaded the flow-through sample onto a Q-Sepharose column and purified M^Pro^ using FPLC by running a linear gradient from 0 to 500 mM NaCl in a buffer (20 mM Tris-HCl, 1 mM DTT, pH 8.0). Fractions eluted from the Q-Sepharose column was concentrated and loaded onto a HiPrep 16/60 Sephacryl S-100 HR column and purified using a buffer containing 20 mM Tris-HCl, 100 mM NaCl, 1 mM DTT, and 1 mM EDTA at pH 7.8. The final purified was concentrated and stored in a −80 °C freezer.

### *In Vitro* M^Pro^ Inhibition Potency Characterizations

We conducted the assay using 20 nM M^Pro^ and 10 μM Sub3 (Figure S2). We dissolved all compounds in DMSO as 10 mM stock solutions. Sub3 was dissolved in DMSO as a 1 mM stock solution and diluted 100 times in the final assay buffer containing 10 mM Na_*x*_H_*y*_PO_4_, 10 mM NaCl, 0.5 mM EDTA, and 1.25% DMSO at pH 7.6. We incubated M^Pro^ and an inhibitor in the final assay buffer for 30 min before adding the substrate to initiate the reaction catalyzed by M^Pro^. The production format was monitored in a fluorescence plate reader with excitation at 336 nm and emission at 490 nm.

### Establishment of 293T Cells Stably Expressing M^Pro^-eGFP

To establish a 293T cell line that stably expresses M^Pro^-eGFP, we packaged lentivirus particles using the pLVX-M^Pro^-eGFP plasmid; detailed preparation was shown in a previous publication.^52^ Briefly, we transfected 293T cells at 90% confluency with three plasmids, including pLVX-M^Pro^-eGFP, pMD2.G, and psPAX2 using 30 mg/mL polyethylenimine. We collected supernatants at 48 and 72 h after transfection separately. We concentrated and collected lentiviral particles from collected supernatant using ultracentrifugation. We then transduced fresh 293T cells using the collected lentivirus particles. After 48 h of transduction, we added puromycin to the culture media to a final concentration of 2 μg/mL. We gradually raised the puromycin concentration 10 μg/mL in 2 weeks. The final stable cells were maintained in media containing 10 μg/mL puromycin.

### Western Blot and DC_50_ (Half-Maximal Degradation Concentration) Analysis

For protein extraction, 5 × 10^5^ cells per well were plated onto 12-well plates and treated with a PROTAC molecule at indicated doses and time duration. We performed protein extraction and Western blot analysis. In a solution containing 50 mM Tris (pH 7.5), 150 mM NaCl, 5 g/mL aprotinin, 1 g/mL pepstatin, 1% Nonidet P-40, 1 mM EDTA, and 0.25% deoxycholate, cells were lysed. In a cold room, protein lysates were sonicated for two min before being centrifuged at maximum speed (14,000 rpm) for 15 min. Using the Bradford reagent (catalog no. 97065-020, VWR), the protein content in the supernatants was measured. After normalizing the protein concentration, the samples were reduced in 4 × Laemmli’s SDS-sample buffer and desaturated at 95 °C for 10 min. Using 10% sodium dodecyl sulfate polyacrylamide gel electrophoresis (SDS-PAGE), an equal quantity of protein samples (20 μg each lane) were separated. Subsequent protein signals were produced using Pierce ECL Western blotting substrate (Thermo Scientific; 32106) and visualized using the ChemiDoc MP Imaging System (Bio-Rad). Data were represented as relative band intensities adjusted to an equal loading control. Antibody against SARS-CoV-2 M^Pro^ was acquired from Thermo Fisher Scientific (cat. no. PA5-116940). β-Actin antibody was purchased from ABCAM (Cat. No. ab124964). Goat Anti-Rabbit IgG H&L (HRP) was acquired from ABCAM as the secondary antibody (Cat. No. ab6721).

Dose–response curves for the tested drugs were obtained in M^Pro^-eGFP 293T stable cells. Various dilutions of the drugs were applied to the 80% confluent cell monolayers and assayed after 48 h to determine the DC_50_ value. β-Actin was used as a loading control in all immunoblot analyses. The normalized protein content in the immunoblots is presented as mean values ± s.e.m. (*n* = 3 biologically independent experiments). Nonlinear regression analysis of GraphPad Prism software (version 8.0) was used to calculate DC_50_ by plotting log compound concentration versus normalized response (variable compounds).

### CRBN Knockout by CRISPR/Cas9 Genomic Editing

To deplete CRBN, CRISPR/Cas9 KO Plasmids, consisting of CRBN-specific 20 nt guide RNA sequences derived from the GeCKO (v2) library, were purchased from Santa Cruz (sc-425519). Briefly, in a 6-well tissue culture plate, 1.5 × 10^5^–2.5 × 10^5^ cells were seeded in 3 mL of antibiotic-free standard growth medium per well, 24 h prior to transfection. Cells were grown to a 40–80% confluency. Initial cell seeding and cell confluency after 24 h are determined based on the rate of cell growth of the cells used for transfection. Healthy and subconfluent cells are required for a successful knockout.

### Viruses

SARS-CoV-2 strains WA.1 (2019-nCoV/USA_WA1/2020) and BA.1 (GISAID EPI_ISL_6640916) were recovered from VeroE6-TMPRSS2 cells by using the established SARS-CoV-2 reverse genetic system (Reference: *Cell Host Microbe***2020** May 13;*27*(5):841–848.e3.; *Emerg. Microbes Infect.***2023** Dec;*12*(1):e2161422.). XBB.1.5/omicron strain 21554–25363 (GenBank entry OX397341.1) was obtained through the World Reference Center for Emerging Viruses and Arboviruses (WRCEVA) at UTMB. A highly attenuated WA.1-derived Δ3678 mGFP SARS-CoV-2 was used as a backbone to incorporate the NSP5 E166A mutation (reference: *Viruses*. **2023** Sep; 15(9):1855.). SARS-CoV-2 infections were performed at the BSL-3 facility at UTMB by personnel equipped with air-purifying respirators.

### Live Virus Antiviral Testing against the SARS-CoV-2 Delta Variant

SARS-CoV-2 delta variant hCoV-19/USA/MD-HP05647/2021 (BEI Resources, NR-55672) was propagated in A549-hACE2 cells (BEI Resources NR-53522) for antiviral testing, at 37 °C in an air-jacketed incubator, with 5% CO_2_ and >90% relative humidity, under BSL-3 conditions at the Texas A&M Global Health Research Complex. A low-dose, multistep growth protocol was used for live virus EC_50_ assays. Briefly, A549-hACE2 cells were cultured in DMEM supplemented with 10% fetal bovine serum overnight. Approximately 5 × 10^4^ A549-hACE2 cells were inoculated by adding 10^3^ infectious units of SARS-CoV-2, as determined by the tissue culture infectious dose 50% (TCID_50_) assay and incubated at 37 °C for 1 h. Cells were then aspirated and rinsed three times with room temperature phosphate-buffered saline, to remove residual inoculum, before replacing DMEM with 10% FBS. Serial 3-fold dilutions of candidate antivirals were made in DMEM with 10% FBS and added to three replicate wells per treatment condition. Infected, treated cells were then cultured for 72 h at 37 °C and 5% CO_2_. At 48 h and 72 h after inoculation, 50 μL of tissue culture medium was removed from each sample for SARS-CoV-2 reverse transcriptase quantitative polymerase chain reaction (RT-qPCR). After 72 h, the cell culture supernatant was removed by aspiration, cells were fixed in 10% formalin, 1× phosphate-buffered saline for at least 30 min, and then stained with crystal violet in order to qualitatively assess the cytopathic effect. EC_50_ values were calculated from the slope and intercept of log-transformed RT-qPCR results, at the point where the linear portion of the transformed dose–response curve showed 50% reduced growth compared to infected, untreated controls. Samples were processed, and RT-qPCR was performed as per protocol established previously.^66^ Samples were diluted 1:1 in 2× Tris-borate-EDTA [TBE] containing 1% Tween-20 and heated at 95 °C for 15 min to lyse and inactivate virions. RT-qPCR screening was performed using the CDC N1 oligonucleotide pair/FAM probe (CDC N1-F, 5′-GACCCCAAAATCAGCGAAAT-3′; CDC N1-R, 5′-TCTGGTTACTGCCAGTTGAATCTG-3′; and Probe CDC N1, 5′ FAM-ACCCCGCATTACGTTTGGTGGACC-BHQ1 3′) and the Luna Universal Probe one-step RT-qPCR kit (catalog no. E3006; New England Biolabs). A 20-μL RT-qPCR mixture contained 7 μL of sample, 0.8 μL each of forward and reverse oligonucleotides (10 μM), 0.4 μL of probe (10 μM), and 11 μL of NEB Luna one-step RT-qPCR 2× master mix. Samples were incubated at 55 °C for 10 min for cDNA synthesis, followed by 95 °C for 1 min (1 cycle) and then 41 cycles of 95 °C for 10 s and 60 °C for 30 s. Genome copies were quantitated relative to quantitative PCR control RNA from heat-inactivated SARS-Related Coronavirus 2, Isolate USA-WA1/2020 (BEI Resources, NR-52347).

### Antiviral Assays against Authentic SARS-CoV-2

A549-hACE2 cells (1.2 × 10^4^) were seeded in each well of a flat-bottom 96-well plate (NUNC) and incubated at 37 °C, 5% CO_2_. The next day, cells were infected with WA.1 (MOI 0.1), BA.1 (MOI 0.3), or XBB.1.5 (MOI 0.1) at 37 °C for 1 h. 2-Fold serial dilutions of compounds were prepared in dimethyl sulfoxide (DMSO). The compounds were further diluted 200-fold in a culture medium containing 2% FBS. After infection, the inoculum was replaced by 100 μL of fresh medium assay containing the diluted compound. At 48 h postinfection, 80 μL per well of supernatants were collected and mixed with 400 μL of Trizol LS (Thermo Fisher Scientific). RNAs were extracted by the Direct-zol RNA Miniprep Plus kit (Zymo Research) and eluted in 50 μL of RNase-free water. RT-qPCR was performed to quantify the viral RNA copies. Briefly, 2 μL of RNA samples were used in a 20 μL reaction system with iTaq SYBR Green One-Step Kit (Bio-Rad) and a pair of primers (CoV19-N2-F: TTACAAACATTGGCCGCAAA; and CoV19-N2-R: GCGCGACATTCCGAAGAA) targeting the N-gene. A standard RT-qPCR was performed on the Applied Biosystems QuantStudio 7 system (Thermo Fisher Scientific) following the manufacturers’ instructions. The relative RNA levels were calculated by normalizing the cycle threshold (CT) values from DMSO controls (the DMSO control was treated as 100%). Two to three independent experiments were performed.

### Antiviral Assays against Δ3678 mGFP SARS-CoV-2

A549-hACE2 cells (1.0 × 10^4^) were seeded in each well of a flat-bottom 96-well plate (NUNC) and incubated at 37 °C, 5% CO_2_. The next day, 2-fold serial dilutions of compounds were prepared in dimethyl sulfoxide (DMSO). The compounds were further diluted 100-fold in a 2% FBS culture medium containing Δ3678 mGFP SARS-CoV-2s (MOI 0.2). 50 μL of the compound–virus mixture was added to each well for infection. At 48 h postinfection, 25 μL of a Hoechst 33342 solution (400-fold diluted in PBS; Thermo Fisher Scientific) was added to each well to counterstain the cell nuclei. After 20 min of incubation at 37 °C, cells were scanned using the CellInsight CX5 high-content screening platform (Thermo Fisher Scientific) with predefined threshold parameters obtained using noninfected and infected cells. The positive cells in each well were counted and normalized to the number of total cells, resulting in the infection rate. The infection rate from each well was finally normalized to the DMSO-treated controls to calculate the relative infectivities. The relative infectivity versus the compound concentration (log10 values) was plotted by using Prism 10. A nonlinear regression method with the log (inhibitor) vs response-variable slope (four parameters) model (bottom and top parameters were constrained to 0 and 100, respectively) was used to determine the compound concentration that inhibits 50% of mGFP Δ3678 infection (defined as EC_50_) in Prism 10. Three independent experiments were performed.

### Cytotoxicity Assay of the PROTAC Degraders

To assess the half-maximal cytotoxic concentration (CC_50_), stock solutions of the tested PROTAC compounds were dissolved in DMSO (10 mM) and diluted further to the working solutions with DMEM. HEK293T cells were seeded in 96-well plates and incubated at 37 °C and 5% CO_2_ for 24 h. The cells were then treated with different concentrations (200, 100, 50, 25, 12.5, 6.25, 3.125, 1.5625, 0.78125, and 0 mM) of the tested compounds in triplicates for 48 h. Cell viability was assessed by the MTT assay to determine CC_50_. 20 mL of MTT (5 mg/mL) was incubated per well for 4 h and then after removing supernatant, 200 mL of DMSO was added per well. The absorbance was recorded at 490 nm to determine the CC_50_. The CC_50_ values were obtained by plotting the normalization % cell viability versus the log_10_ sample concentration.

### *In Vitro* Metabolic Stability in Human Liver Microsomes

This metabolic stability measurements were based on previous publications and modified as described below.^67^ The metabolic stability profile of the inhibitor, including CL_int,pred_ and *in vitrot*_1/2_ was determined by the estimation of the remaining compound concentration after incubation with human liver microsome, NADPH (cofactor), EDTA, and MgCl_2_ in a 0.1 M phosphate buffer (pH 7.4). 5 μM of each inhibitor was preincubated with 40 μL of human liver microsome (0.5 mg/mL) in 0.1 M phosphate buffer (pH 7.4) at 37 °C for 5 min to set optimal conditions for metabolic reactions. After preincubation, NADPH (5 mM, 10 μL) or 0.1 M PB (10 μL) was added to initiate metabolic reaction at 37 °C. The reactions were conducted in triplicate. At 0, 5, 15, 30, 45, and 60 min, 200 μL of acetonitrile (with internal standard Diclofenac, 10 μg/mL) was added in order to quench the reaction. The samples were subjected to centrifugation at 4 °C for 20 min at 4000 rpm. Then, 50 μL of clear supernatants were analyzed by high-performance liquid chromatography-tandem mass spectrometry (HPLC-MS/MS). The percentage of test compound remaining was determined by the following formula: % remaining = (area at *t*_*x*_/average area at *t*_0_) × 100. The half-life (*t*_1/2_) was calculated using the slope (*k*) of the log–linear regression from the % remaining parent compound versus time (min) relationship: *t*_1/2_ (min) = −ln 2/*k*. CL_int,pred_ (mL/min/kg) was calculated through the following formula CL_int, pred_ = (0.693/*t*_1/2_) × (1/(microsomal protein concentration (0.5 mg/mL))) × scaling Factor (1254.16 for human liver microsome).

### Synthesis of MPDs

All reagents and solvents for the synthesis were purchased from commercial sources and used without purification. All glassware was flame-dried prior to use. Thin-layer chromatography (TLC) was carried out on aluminum plates coated with 60 F254 silica gel. TLC plates were visualized under UV light (254 or 365 nm) or stained with 5% phosphomolybdic acid. Normal phase column chromatography was carried out using a Yamazen Small Flash AKROS system. NMR spectra were recorded on a Bruker AVANCE Neo 400 MHz spectrometer in specified deuterated solvents. The purity of the final MPDs was assessed by LC-MS to confirm >95% purity. Analytical liquid chromatography–mass spectrometry was performed on a PHENOMENEX C18 Column (150 mm × 2.00 mm 5 μm, gradient from 10% to 100% B [A = 10 mmol/L HCOONH_4_/H_2_O, B = MeOH], flow rate: 0.3 mL/min) using a Thermo Scientific Ultimate 3000 with a UV-detector (detection at 215 nm), equipped with a Thermo Scientific Orbitrap Q Exactive Focus System.

## Supporting Information Available

Structures of MPD1-10; cytotoxicity of the MPD1-MPD3 in 293T cells; flow cytometry analysis of the potency of MPD2 in degrading M^Pro^; synthesis of MPD1-MPD10; ^1^H NMR, ^13^C NMR spectra; and HPLC (PDF)
Molecular formula strings (CSV)

## References

[ref1] Perlman S.; Netland J. Coronaviruses post-SARS: update on replication and pathogenesis. Nat. Rev. Microbiol. 2009, 7 (6), 439–450. 10.1038/nrmicro2147. 19430490 PMC2830095

[ref2] Principi N.; Bosis S.; Esposito S. Effects of coronavirus infections in children. Emerging Infect. Dis. 2010, 16 (2), 183–188. 10.3201/eid1602.090469. PMC2957994 20113545

[ref3] Adachi S.; Koma T.; Doi N.; Nomaguchi M.; Adachi A. Commentary: Origin and evolution of pathogenic coronaviruses. Front. Immunol. 2020, 11, 811 10.3389/fimmu.2020.00811. 32373134 PMC7187924

[ref4] Cui J.; Li F.; Shi Z. L. Origin and evolution of pathogenic coronaviruses. Nat. Rev. Microbiol. 2019, 17 (3), 181–192. 10.1038/s41579-018-0118-9. 30531947 PMC7097006

[ref5] Fung T. S.; Liu D. X. Human Coronavirus: Host-Pathogen Interaction. Annu. Rev. Microbiol. 2019, 73, 529–557. 10.1146/annurev-micro-020518-115759. 31226023

[ref6] McIntosh K.; Dees J. H.; Becker W. B.; Kapikian A. Z.; Chanock R. M. Recovery in tracheal organ cultures of novel viruses from patients with respiratory disease. Proc. Natl. Acad. Sci. U.S.A. 1967, 57 (4), 933–940. 10.1073/pnas.57.4.933. 5231356 PMC224637

[ref7] Hamre D.; Procknow J. J. A new virus isolated from the human respiratory tract. Exp. Biol. Med. 1966, 121 (1), 190–193. 10.3181/00379727-121-30734. 4285768

[ref8] Vijayanand P.; Wilkins E.; Woodhead M. Severe acute respiratory syndrome (SARS): a review. Clin. Med. 2004, 4 (2), 152–160. 10.7861/clinmedicine.4-2-152. PMC4954004 15139736

[ref9] Ramadan N.; Shaib H. Middle East respiratory syndrome coronavirus (MERS-CoV): A review. Germs 2019, 9 (1), 35–42. 10.18683/germs.2019.1155. 31119115 PMC6446491

[ref10] Zhu N.; Zhang D.; Wang W.; Li X.; Yang B.; Song J.; Zhao X.; Huang B.; Shi W.; Lu R.; Niu P.; Zhan F.; Ma X.; Wang D.; Xu W.; Wu G.; Gao G. F.; Tan W. A Novel Coronavirus from Patients with Pneumonia in China, 2019. N. Engl. J. Med. 2020, 382 (8), 727–733. 10.1056/NEJMoa2001017. 31978945 PMC7092803

[ref11] Khailany R. A.; Safdar M.; Ozaslan M. Genomic characterization of a novel SARS-CoV-2. Gene Rep. 2020, 19, 100682 10.1016/j.genrep.2020.100682. 32300673 PMC7161481

[ref12] Kim D.; Lee J.-Y.; Yang J.-S.; Kim J. W.; Kim V. N.; Chang H. The Architecture of SARS-CoV-2 Transcriptome. Cell 2020, 181 (4), 914–921.e10. 10.1016/j.cell.2020.04.011. 32330414 PMC7179501

[ref13] Chen Y.; Liu Q.; Guo D. Emerging coronaviruses: Genome structure, replication, and pathogenesis. J. Med. Virol. 2020, 92 (4), 418–423. 10.1002/jmv.25681. 31967327 PMC7167049

[ref14] Cui J.; Li F.; Shi Z.-L. Origin and evolution of pathogenic coronaviruses. Nat. Rev. Microbiol. 2019, 17 (3), 181–192. 10.1038/s41579-018-0118-9. 30531947 PMC7097006

[ref15] Wong G.; Liu W.; Liu Y.; Zhou B.; Bi Y.; Gao G. F. MERS, SARS, and Ebola: The Role of Super-Spreaders in Infectious Disease. Cell Host Microbe 2015, 18 (4), 398–401. 10.1016/j.chom.2015.09.013. 26468744 PMC7128246

[ref16] Phan T. Genetic diversity and evolution of SARS-CoV-2. Infect. Genet. Evol. 2020, 81, 104260 10.1016/j.meegid.2020.104260. 32092483 PMC7106203

[ref17] Chen Y. W.; Yiu C.-P. B.; Wong K.-Y. Prediction of the SARS-CoV-2 (2019-nCoV) 3C-like protease (3CLpro) structure: virtual screening reveals velpatasvir, ledipasvir, and other drug repurposing candidates. F1000Research 2020, 9, 129 10.12688/f1000research.22457.2. 32194944 PMC7062204

[ref18] Ullrich S.; Nitsche C. The SARS-CoV-2 main protease as drug target. Bioorg. Med. Chem. Lett. 2020, 30 (17), 127377 10.1016/j.bmcl.2020.127377. 32738988 PMC7331567

[ref19] Akaji K.; Konno H.; Mitsui H.; Teruya K.; Shimamoto Y.; Hattori Y.; Ozaki T.; Kusunoki M.; Sanjoh A. Structure-Based Design, Synthesis, and Evaluation of Peptide-Mimetic SARS 3CL Protease Inhibitors. J. Med. Chem. 2011, 54 (23), 7962–7973. 10.1021/jm200870n. 22014094

[ref20] Liu Y.; Liang C.; Xin L.; Ren X.; Tian L.; Ju X.; Li H.; Wang Y.; Zhao Q.; Liu H.; Cao W.; Xie X.; Zhang D.; Wang Y.; Jian Y. The development of Coronavirus 3C-Like protease (3CLpro) inhibitors from 2010 to 2020. Eur. J. Med. Chem. 2020, 206, 112711 10.1016/j.ejmech.2020.112711. 32810751 PMC7409838

[ref21] Anand K.; Ziebuhr J.; Wadhwani P.; Mesters J. R.; Hilgenfeld R. Coronavirus Main Proteinase (3CLpro) Structure: Basis for Design of Anti-SARS Drugs. Science 2003, 300 (5626), 1763 10.1126/science.1085658. 12746549

[ref22] Owen D. R.; Allerton C. M. N.; Anderson A. S.; Aschenbrenner L.; Avery M.; Berritt S.; Boras B.; Cardin R. D.; Carlo A.; Coffman K. J.; Dantonio A.; Di L.; Eng H.; Ferre R.; Gajiwala K. S.; Gibson S. A.; Greasley S. E.; Hurst B. L.; Kadar E. P.; Kalgutkar A. S.; Lee J. C.; Lee J.; Liu W.; Mason S. W.; Noell S.; Novak J. J.; Obach R. S.; Ogilvie K.; Patel N. C.; Pettersson M.; Rai D. K.; Reese M. R.; Sammons M. F.; Sathish J. G.; Singh R. S. P.; Steppan C. M.; Stewart A. E.; Tuttle J. B.; Updyke L.; Verhoest P. R.; Wei L.; Yang Q.; Zhu Y. An oral SARS-CoV-2 Mpro inhibitor clinical candidate for the treatment of COVID-19. Science 2021, 374 (6575), 1586–1593. 10.1126/science.abl4784. 34726479

[ref23] Yang K. S.; Leeuwon S. Z.; Xu S.; Liu W. R. Evolutionary and Structural Insights about Potential SARS-CoV-2 Evasion of Nirmatrelvir. J. Med. Chem. 2022, 65 (13), 8686–8698. 10.1021/acs.jmedchem.2c00404. 35731933 PMC9236210

[ref24] Chatterjee S.; Bhattacharya M.; Dhama K.; Lee S.-S.; Chakraborty C. Resistance to nirmatrelvir due to mutations in the Mpro in the subvariants of SARS-CoV-2 Omicron: Another concern?. Mol. Ther. Nucleic Acids 2023, 32, 263–266. 10.1016/j.omtn.2023.03.013. 37041859 PMC10078092

[ref25] Hu Y.; Lewandowski E. M.; Tan H.; Zhang X.; Morgan R. T.; Zhang X.; Jacobs L. M. C.; Butler S. G.; Gongora M. V.; Choy J.; Deng X.; Chen Y.; Wang J. Naturally Occurring Mutations of SARS-CoV-2 Main Protease Confer Drug Resistance to Nirmatrelvir. ACS Cent. Sci. 2023, 9 (8), 1658–1669. 10.1021/acscentsci.3c00538. 37637734 PMC10451032

[ref26] Burslem G. M.; Crews C. M. Proteolysis-Targeting Chimeras as Therapeutics and Tools for Biological Discovery. Cell 2020, 181 (1), 102–114. 10.1016/j.cell.2019.11.031. 31955850 PMC7319047

[ref27] Chamberlain P. P.; Hamann L. G. Development of targeted protein degradation therapeutics. Nat. Chem. Biol. 2019, 15 (10), 937–944. 10.1038/s41589-019-0362-y. 31527835

[ref28] Gao H.; Sun X.; Rao Y. PROTAC Technology: Opportunities and Challenges. ACS Med. Chem. Lett. 2020, 11 (3), 237–240. 10.1021/acsmedchemlett.9b00597. 32184950 PMC7073876

[ref29] Konstantinidou M.; Li J.; Zhang B.; Wang Z.; Shaabani S.; Ter Brake F.; Essa K.; Dömling A. PROTACs– a game-changing technology. Expert Opin. Drug Discovery 2019, 14 (12), 1255–1268. 10.1080/17460441.2019.1659242. PMC7008130 31538491

[ref30] Nalawansha D. A.; Crews C. M. PROTACs: An Emerging Therapeutic Modality in Precision Medicine. Cell Chem. Biol. 2020, 27 (8), 998–1014. 10.1016/j.chembiol.2020.07.020. 32795419 PMC9424844

[ref31] Paiva S.-L.; Crews C. M. Targeted protein degradation: elements of PROTAC design. Curr. Opin. Chem. Biol. 2019, 50, 111–119. 10.1016/j.cbpa.2019.02.022. 31004963 PMC6930012

[ref32] Jang J.; To C.; De Clercq D. J. H.; Park E.; Ponthier C. M.; Shin B. H.; Mushajiang M.; Nowak R. P.; Fischer E. S.; Eck M. J.; Jänne P. A.; Gray N. S. Mutant-Selective Allosteric EGFR Degraders are Effective Against a Broad Range of Drug-Resistant Mutations. Angew. Chem. Int. Ed. 2020, 59 (34), 14481–14489. 10.1002/anie.202003500. PMC7686272 32510788

[ref33] Shimokawa K.; Shibata N.; Sameshima T.; Miyamoto N.; Ujikawa O.; Nara H.; Ohoka N.; Hattori T.; Cho N.; Naito M. Targeting the Allosteric Site of Oncoprotein BCR-ABL as an Alternative Strategy for Effective Target Protein Degradation. ACS Med. Chem. Lett. 2017, 8 (10), 1042–1047. 10.1021/acsmedchemlett.7b00247. 29057048 PMC5641955

[ref34] Bondeson D. P.; Mares A.; Smith I. E. D.; Ko E.; Campos S.; Miah A. H.; Mulholland K. E.; Routly N.; Buckley D. L.; Gustafson J. L.; Zinn N.; Grandi P.; Shimamura S.; Bergamini G.; Faelth-Savitski M.; Bantscheff M.; Cox C.; Gordon D. A.; Willard R. R.; Flanagan J. J.; Casillas L. N.; Votta B. J.; den Besten W.; Famm K.; Kruidenier L.; Carter P. S.; Harling J. D.; Churcher I.; Crews C. M. Catalytic in vivo protein knockdown by small-molecule PROTACs. Nat. Chem. Biol. 2015, 11 (8), 611–617. 10.1038/nchembio.1858. 26075522 PMC4629852

[ref35] Burslem G. M.; Smith B. E.; Lai A. C.; Jaime-Figueroa S.; McQuaid D. C.; Bondeson D. P.; Toure M.; Dong H.; Qian Y.; Wang J.; Crew A. P.; Hines J.; Crews C. M. The Advantages of Targeted Protein Degradation Over Inhibition: An RTK Case Study. Cell Chem. Biol. 2018, 25 (1), 67–77.e3. 10.1016/j.chembiol.2017.09.009. 29129716 PMC5831399

[ref36] Huang H.-T.; Dobrovolsky D.; Paulk J.; Yang G.; Weisberg E. L.; Doctor Z. M.; Buckley D. L.; Cho J.-H.; Ko E.; Jang J.; Shi K.; Choi H. G.; Griffin J. D.; Li Y.; Treon S. P.; Fischer E. S.; Bradner J. E.; Tan L.; Gray N. S. A Chemoproteomic Approach to Query the Degradable Kinome Using a Multi-kinase Degrader. Cell Chem. Biol. 2018, 25 (1), 88–99.e6. 10.1016/j.chembiol.2017.10.005. 29129717 PMC6427047

[ref37] Gadd M. S.; Testa A.; Lucas X.; Chan K.-H.; Chen W.; Lamont D. J.; Zengerle M.; Ciulli A. Structural basis of PROTAC cooperative recognition for selective protein degradation. Nat. Chem. Biol. 2017, 13 (5), 514–521. 10.1038/nchembio.2329. 28288108 PMC5392356

[ref38] Jiang B.; Wang E. S.; Donovan K. A.; Liang Y.; Fischer E. S.; Zhang T.; Gray N. S. Development of Dual and Selective Degraders of Cyclin-Dependent Kinases 4 and 6. Angew. Chem. Int. Ed. 2019, 58 (19), 6321–6326. 10.1002/anie.201901336. PMC7678623 30802347

[ref39] Smith B. E.; Wang S. L.; Jaime-Figueroa S.; Harbin A.; Wang J.; Hamman B. D.; Crews C. M. Differential PROTAC substrate specificity dictated by orientation of recruited E3 ligase. Nat. Commun. 2019, 10 (1), 131 10.1038/s41467-018-08027-7. 30631068 PMC6328587

[ref40] Bondeson D. P.; Smith B. E.; Burslem G. M.; Buhimschi A. D.; Hines J.; Jaime-Figueroa S.; Wang J.; Hamman B. D.; Ishchenko A.; Crews C. M. Lessons in PROTAC Design from Selective Degradation with a Promiscuous Warhead. Cell Chem. Biol. 2018, 25 (1), 78–87.e5. 10.1016/j.chembiol.2017.09.010. 29129718 PMC5777153

[ref41] Bai L.; Zhou H.; Xu R.; Zhao Y.; Chinnaswamy K.; McEachern D.; Chen J.; Yang C.-Y.; Liu Z.; Wang M.; Liu L.; Jiang H.; Wen B.; Kumar P.; Meagher J. L.; Sun D.; Stuckey J. A.; Wang S. A Potent and Selective Small-Molecule Degrader of STAT3 Achieves Complete Tumor Regression In Vivo. Cancer Cell 2019, 36 (5), 498–511.e17. 10.1016/j.ccell.2019.10.002. 31715132 PMC6880868

[ref42] Leiser D.; Pochon B.; Blank-Liss W.; Francica P.; Glück A. A.; Aebersold D. M.; Zimmer Y.; Medová M. Targeting of the MET receptor tyrosine kinase by small molecule inhibitors leads to MET accumulation by impairing the receptor downregulation. FEBS Lett. 2014, 588 (5), 653–658. 10.1016/j.febslet.2013.12.025. 24440350

[ref43] de Wispelaere M.; Du G.; Donovan K. A.; Zhang T.; Eleuteri N. A.; Yuan J. C.; Kalabathula J.; Nowak R. P.; Fischer E. S.; Gray N. S.; Yang P. L. Small molecule degraders of the hepatitis C virus protease reduce susceptibility to resistance mutations. Nat. Commun. 2019, 10 (1), 3468 10.1038/s41467-019-11429-w. 31371704 PMC6672008

[ref44] Guo W.-H.; Qi X.; Yu X.; Liu Y.; Chung C.-I.; Bai F.; Lin X.; Lu D.; Wang L.; Chen J.; Su L. H.; Nomie K. J.; Li F.; Wang M. C.; Shu X.; Onuchic J. N.; Woyach J. A.; Wang M. L.; Wang J. Enhancing intracellular accumulation and target engagement of PROTACs with reversible covalent chemistry. Nat. Commun. 2020, 11 (1), 4268 10.1038/s41467-020-17997-6. 32848159 PMC7450057

[ref45] Martinez-Ortiz W.; Zhou M.-M. Could PROTACs Protect Us From COVID-19?. Drug Discovery Today 2020, 25 (11), 1894–1896. 10.1016/j.drudis.2020.08.007. 32889063 PMC7462587

[ref46] Espinoza-Chávez R. M.; Salerno A.; Liuzzi A.; Ilari A.; Milelli A.; Uliassi E.; Bolognesi M. L. Targeted Protein Degradation for Infectious Diseases: from Basic Biology to Drug Discovery. ACS Bio Med. Chem. Au 2023, 3 (1), 32–45. 10.1021/acsbiomedchemau.2c00063. PMC10125329 37101607

[ref47] Liang J.; Wu Y.; Lan K.; Dong C.; Wu S.; Li S.; Zhou H.-B. Antiviral PROTACs: Opportunity borne with challenge. Cell Insight 2023, 2 (3), 100092 10.1016/j.cellin.2023.100092. 37398636 PMC10308200

[ref48] Mukerjee N.; Ghosh A. Revolutionizing viral disease treatment: PROTACs therapy could be the ultimate weapon of the future. J. Med. Virol. 2023, 95 (8), e28981 10.1002/jmv.28981. 37515471

[ref49] Sang X.; Wang J.; Zhou J.; Xu Y.; An J.; Warshel A.; Huang Z. A Chemical Strategy for the Degradation of the Main Protease of SARS-CoV-2 in Cells. J. Am. Chem. Soc. 2023, 145 (50), 27248–27253. 10.1021/jacs.3c12678. 38064654

[ref50] Yang K. S.; Ma X. R.; Ma Y.; Alugubelli Y. R.; Scott D. A.; Vatansever E. C.; Drelich A. K.; Sankaran B.; Geng Z. Z.; Blankenship L. R.; Ward H. E.; Sheng Y. J.; Hsu J. C.; Kratch K. C.; Zhao B.; Hayatshahi H. S.; Liu J.; Li P.; Fierke C. A.; Tseng C. K.; Xu S.; Liu W. R. A Quick Route to Multiple Highly Potent SARS-CoV-2 Main Protease Inhibitors. ChemMedChem 2021, 16 (6), 942–948. 10.1002/cmdc.202000924. 33283984 PMC7979488

[ref51] Pillaiyar T.; Manickam M.; Namasivayam V.; Hayashi Y.; Jung S.-H. An Overview of Severe Acute Respiratory Syndrome–Coronavirus (SARS-CoV) 3CL Protease Inhibitors: Peptidomimetics and Small Molecule Chemotherapy. J. Med. Chem. 2016, 59 (14), 6595–6628. 10.1021/acs.jmedchem.5b01461. 26878082 PMC7075650

[ref52] Cao W.; Cho C.-C. D.; Geng Z. Z.; Shaabani N.; Ma X. R.; Vatansever E. C.; Alugubelli Y. R.; Ma Y.; Chaki S. P.; Ellenburg W. H.; Yang K. S.; Qiao Y.; Allen R.; Neuman B. W.; Ji H.; Xu S.; Liu W. R. Evaluation of SARS-CoV-2 Main Protease Inhibitors Using a Novel Cell-Based Assay. ACS Cent. Sci. 2022, 8 (2), 192–204. 10.1021/acscentsci.1c00910. 35229034 PMC8848508

[ref53] Alugubelli Y. R.; Geng Z. Z.; Yang K. S.; Shaabani N.; Khatua K.; Ma X. R.; Vatansever E. C.; Cho C. C.; Ma Y.; Xiao J.; Blankenship L. R.; Yu G.; Sankaran B.; Li P.; Allen R.; Ji H.; Xu S.; Liu W. R. A systematic exploration of boceprevir-based main protease inhibitors as SARS-CoV-2 antivirals. Eur. J. Med. Chem. 2022, 240, 114596 10.1016/j.ejmech.2022.114596. 35839690 PMC9264725

[ref54] Fu L.; Ye F.; Feng Y.; Yu F.; Wang Q.; Wu Y.; Zhao C.; Sun H.; Huang B.; Niu P.; Song H.; Shi Y.; Li X.; Tan W.; Qi J.; Gao G. F. Both Boceprevir and GC376 efficaciously inhibit SARS-CoV-2 by targeting its main protease. Nat. Commun. 2020, 11 (1), 4417 10.1038/s41467-020-18233-x. 32887884 PMC7474075

[ref55] Ma C.; Sacco M. D.; Hurst B.; Townsend J. A.; Hu Y.; Szeto T.; Zhang X.; Tarbet B.; Marty M. T.; Chen Y.; Wang J. Boceprevir, GC-376, and calpain inhibitors II, XII inhibit SARS-CoV-2 viral replication by targeting the viral main protease. Cell Res. 2020, 30 (8), 678–692. 10.1038/s41422-020-0356-z. 32541865 PMC7294525

[ref56] Oerlemans R.; Ruiz-Moreno A. J.; Cong Y.; Dinesh Kumar N.; Velasco-Velazquez M. A.; Neochoritis C. G.; Smith J.; Reggiori F.; Groves M. R.; Domling A. Repurposing the HCV NS3–4A protease drug boceprevir as COVID-19 therapeutics. Rsc Med. Chem. 2021, 12 (3), 370–379. 10.1039/D0MD00367K. PMC8130630 34041486

[ref57] Gabizon R.; Shraga A.; Gehrtz P.; Livnah E.; Shorer Y.; Gurwicz N.; Avram L.; Unger T.; Aharoni H.; Albeck S.; Brandis A.; Shulman Z.; Katz B.-Z.; Herishanu Y.; London N. Efficient Targeted Degradation via Reversible and Irreversible Covalent PROTACs. J. Am. Chem. Soc. 2020, 142 (27), 11734–11742. 10.1021/jacs.9b13907. 32369353 PMC7349657

[ref58] Kiely-Collins H.; Winter G. E.; Bernardes G. J. L. The role of reversible and irreversible covalent chemistry in targeted protein degradation. Cell Chem. Biol. 2021, 28 (7), 952–968. 10.1016/j.chembiol.2021.03.005. 33789091

[ref59] Lu D.; Yu X.; Lin H.; Cheng R.; Monroy E. Y.; Qi X.; Wang M. C.; Wang J. Applications of covalent chemistry in targeted protein degradation. Chem. Soc. Rev. 2022, 51 (22), 9243–9261. 10.1039/D2CS00362G. 36285735 PMC9669245

[ref60] Yuan M.; Chu Y.; Duan Y. Reversible Covalent PROTACs: Novel and Efficient Targeted Degradation Strategy. Front. Chem. 2021, 9, 691093 10.3389/fchem.2021.691093. 34291036 PMC8287302

[ref61] Lopez-Girona A.; Mendy D.; Ito T.; Miller K.; Gandhi A. K.; Kang J.; Karasawa S.; Carmel G.; Jackson P.; Abbasian M.; Mahmoudi A.; Cathers B.; Rychak E.; Gaidarova S.; Chen R.; Schafer P. H.; Handa H.; Daniel T. O.; Evans J. F.; Chopra R. Cereblon is a direct protein target for immunomodulatory and antiproliferative activities of lenalidomide and pomalidomide. Leukemia 2012, 26 (11), 2326–2335. 10.1038/leu.2012.119. 22552008 PMC3496085

[ref62] Vatansever E. C.; Yang K. S.; Drelich A. K.; Kratch K. C.; Cho C.-C.; Kempaiah K. R.; Hsu J. C.; Mellott D. M.; Xu S.; Tseng C.-T. K.; Liu W. R. Bepridil is potent against SARS-CoV-2 in vitro. Proc. Natl. Acad. Sci. U.S.A. 2021, 118 (10), e2012201118 10.1073/pnas.2012201118. 33597253 PMC7958448

[ref63] Kumar P.; Nagarajan A.; Uchil P. D. Analysis of Cell Viability by the MTT Assay. Cold Spring Harbor Protoc. 2018, 469–471. 10.1101/pdb.prot095505. 29858338

[ref64] Kiso M.; Yamayoshi S.; Iida S.; Furusawa Y.; Hirata Y.; Uraki R.; Imai M.; Suzuki T.; Kawaoka Y. In vitro and in vivo characterization of SARS-CoV-2 resistance to ensitrelvir. Nat. Commun. 2023, 14 (1), 4231 10.1038/s41467-023-40018-1. 37454219 PMC10349878

[ref65] Zou J.; Kurhade C.; Chang H. C.; Hu Y.; Meza J. A.; Beaver D.; Trinh K.; Omlid J.; Elghetany B.; Desai R.; McCaffrey P.; Garcia J. D.; Shi P.-Y.; Ren P.; Xie X. An Integrated Research–Clinical BSL-2 Platform for a Live SARS-CoV-2 Neutralization Assay. Viruses 2023, 15 (9), 1855 10.3390/v15091855. 37766263 PMC10536566

[ref66] Chaki S. P.; Kahl-McDonagh M. M.; Neuman B. W.; Zuelke K. A. Receptor-Binding-Motif-Targeted Sanger Sequencing: a Quick and Cost-Effective Strategy for Molecular Surveillance of SARS-CoV-2 Variants. Microbiol. Spectrum 2022, 10 (3), e0066522 10.1128/spectrum.00665-22. PMC9241651 35638906

[ref67] Attwa M. W.; Abdelhameed A. S.; Alsaif N. A.; Kadi A. A.; AlRabiah H. A validated LC-MS/MS analytical method for the quantification of pemigatinib: metabolic stability evaluation in human liver microsomes. RSC Adv. 2022, 12 (31), 20387–20394. 10.1039/D2RA02885A. 35919584 PMC9277622

